# Safety profile of complement C5 inhibitors and FcRn inhibitors in the treatment of myasthenia gravis: analysis of the FAERS database and disease-gene interaction network

**DOI:** 10.3389/fimmu.2025.1667249

**Published:** 2025-10-08

**Authors:** Luqiong Wang, Jiaojiao Chen, Huixiang Li, Lin Wang, Feiyu Liu, Xiaoli Jiang

**Affiliations:** Department of Pharmacy, Yantai Yuhuangding Hospital Affiliated to Qingdao University, Yantai, Shandong, China

**Keywords:** FAERS database, adverse reactions, FcRn inhibitor, complement C5 inhibitor, eculizumab, ravulizumab, zilucoplan, efgartigimod

## Abstract

**Objective:**

To integrate pharmacovigilance and network pharmacology methods for a comprehensive analysis of the potential adverse reactions of complement C5 inhibitors (eculizumab, ravulizumab, zilucoplan) and neonatal Fc receptor (FcRn) inhibitors (efgartigimod, rozanolixizumab), and to explore their toxicity mechanisms, thereby providing a reference for rapidly understanding the safety of these two novel classes of biologics in the treatment of myasthenia gravis (MG).

**Methods:**

We extracted adverse event (AE) reports for these five drugs from the FDA Adverse Event Reporting System (FAERS) database, limited to the period since their FDA approval for the treatment of MG. Reports were further restricted to those where the drug was listed as the primary suspect (PS) and the indication (INDI) was “MG”. Signal detection was performed using the Reporting Odds Ratio (ROR) method, the UK Medicines and Healthcare products Regulatory Agency (MHRA) method, and the Bayesian Confidence Propagation Neural Network (BCPNN) method. Additionally, network pharmacology was employed to analyze the toxicity mechanisms of the system organ categories (SOCs) specifically associated with complement C5 inhibitors and FcRn inhibitors.

**Results:**

Signal detection of AE reports associated with these five drugs revealed previously unlabeled positive signals, including: eculizumab (gastric cancer, embolic stroke), ravulizumab (psoriatic arthropathy, hypoacusis, peripheral vascular disorders), zilucoplan (weight increased, weight decreased), efgartigimod (metastases to liver, hepatic failure, nephrolithiasis, dysuria, Prostatitis, prostate cancer, Angina pectoris, congestive cardiac failure) and rozanolixizumab (vomiting, dyspepsia). However, the gastric cancer, liver metastasis and prostate cancer were reported within the first 30 days, causal associations cannot be established based on the data presented. Potential toxicity analysis was conducted on noteworthy SOCs for complement C5 inhibitors and FcRn inhibitors, revealing key targets and pathways.

**Conclusion:**

This study elucidated the safety profiles of complement C5 inhibitors and FcRn inhibitors in clinical practice through pharmacovigilance analysis, confirming known adverse reactions and identifying several previously unreported ones. Furthermore, network pharmacology analysis revealed potential mechanisms underlying these adverse reactions. These findings provide valuable insights for monitoring and managing risks during treatment with two novel classes of biologics.

## 1 Introduction

Myasthenia gravis, a rare chronic disorder, has shown increasing global prevalence and incidence over the past decades (1). Since DW Smithers first proposed MG as an autoimmune disease in 1958, its treatment has primarily relied on acetylcholinesterase inhibitors for symptomatic relief, combined with conventional immunotherapy involving low-dose corticosteroids and non-steroidal immunosuppressants (2–5). For generalized MG or cases complicated by thymoma, thymectomy is often employed, while intravenous immunoglobulins or plasma exchange are used during myasthenic crises or acute exacerbations (6, 7). However, the systemic adverse effects of corticosteroids and immunosuppressants (8, 9), along with the invasive nature of plasma exchange and thymectomy (7, 10), pose significant safety challenges in MG management. These limitations underscore the need for safer therapeutic alternatives.

Until 2017, eculizumab was authorized for the treatment of adult patients with acetylcholine receptor (AChR) antibody-positive generalized myasthenia gravis (gMG) (11). As the first complement C5 inhibitor for MG, it specifically binds to C5 protein, preventing its cleavage and subsequent formation of the membrane attack complex, thereby protecting the postsynaptic membrane (12). In March 2025, the FDA expanded the approved use of eculizumab to include children aged 6 and above based on a 26-week RCT study involving 11 pediatric patients aged 12 to 17 years (13). This marked a significant milestone in the treatment of MG. Another complement C5 inhibitor, ravulizumab, was approved by the European Medicines Agency (EMA) in September 2022 for adult patients with AChR antibody-positive gMG (14). As the first and only long-acting complement C5 inhibitor approved for gMG, it significantly improved patients’ quality of life by reducing dosing frequency compared to eculizumab (15). In October 2023, the FDA approved zilucoplan, a C5-targeting cyclic peptide inhibitor administered via subcutaneous injection, offering complete inhibition of the complement pathway and presenting a novel therapeutic option for gMG patients (8, 16). This advancement may address limitations of existing complement C5 inhibitors, such as restricted administration routes and suboptimal efficacy in some patients.

With advances in biologic therapies, the treatment of MG has achieved new breakthroughs. In December 2021, efgartigimod became the first FcRn inhibitor approved worldwide by the FDA (17). Unlike complement C5 inhibitors, this drug was immediately indicated for the treatment of gMG in adult patients with anti-AChR antibody positivity. Following a positive vote from the EMA, the European Union approved efgartigimod alfa in August 2022 as an additive therapy to standard treatment for AChR antibody-positive adult gMG patients (18). Another FcRn inhibitor, rozanolixizumab, was approved in 2023 for adult patients with AChR and musclespecific kinase (MuSK) antibody-positive gMG. These novel targeted therapies have made significant contributions to the treatment of MG (19).

As novel therapeutic agents for MG, complement C5 and FcRn inhibitors have only been on the market for a short period and have not yet been widely adopted in many countries and regions. Consequently, there remains limited clinical experience with these drugs, and both physicians and patients have incomplete understanding of their safety profiles. Apart from the well-known adverse reactions mentioned in the labels (such as meningococcal infection caused by complement C5 inhibitors, and headache caused by FcRn inhibitors), there may still be unrecognized adverse reactions. In this study, we employed FAERS data mining to identify potential AE signals associated with these drugs. Through restricting indication, we were able to detect stronger AE signals with greater precision. Furthermore, we utilized network pharmacology approaches to investigate the potential mechanisms underlying AE occurrences related to these two novel classes of biologics. Our findings aim to provide valuable references for optimizing drug selection in MG treatment.

## 2 Methods

### 2.1 Data source

We conducted a retrospective pharmacovigilance study using data from the FAERS database (https://fis.fda.gov/extensions/FPD-QDE-FAERS/FPD-QDE-FAERS.html). This publicly available database contains spontaneous AE reports submitted by consumers, healthcare professionals and manufacturers. We downloaded raw ASCII data packages from 2017 Q4 to 2025 Q1 and performed statistical analyses for each of these five drugs starting from their respective approval dates for MG treatment. The raw data files consist of seven datasets, including demographic and management information (DEMO), drug information (DRUG), adverse reaction information (REAC), outcome information (OUTC), reporting source information (RPSR), treatment start and end date (THER), and drug administration indications (INDI).

### 2.2 Data extraction

According to the recommendations of the FDA guidelines (20), the data were preprocessed using the SAS software. For reports with the same case identifier (CASEID), the report with the latest FDA reception date (FDA_DT) was retained; when the values of CASEID and FDA_DT matched, the report with the highest PRIMARYID (the unique identifier assigned to each report) was retained.

**Figure 1:**
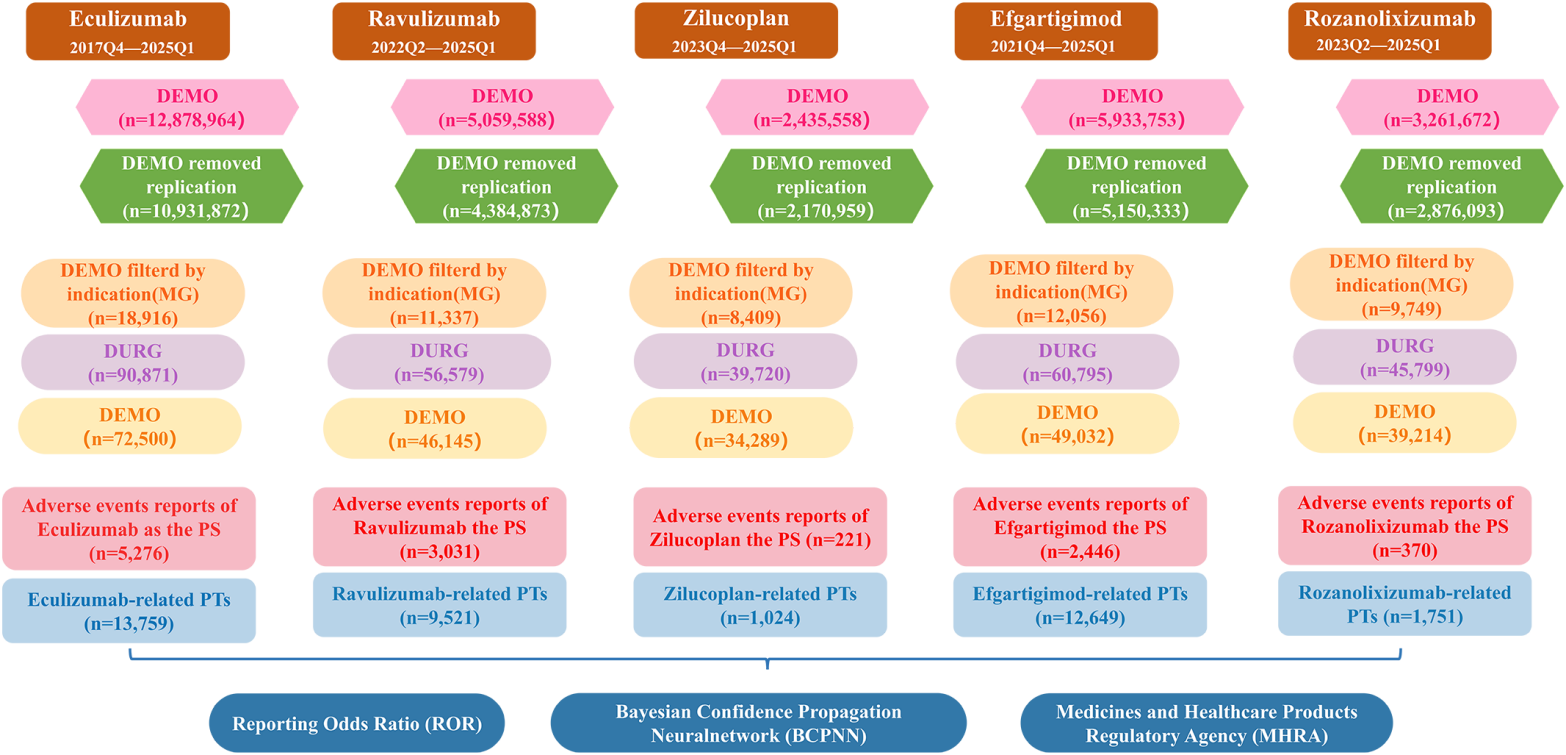
A flowchart illustrating the process of adverse event analysis using the FAERS database. DEMO, demographic and management information; MG, myasthenia gravis; DRUG, drug information; PS, primary suspect; PTs, preferred terms.

This study included a total of five drugs, among which eculizumab, ravulizumab and zilucoplan are complement C5 inhibitors, efgartigimod and rozanolixizumab are FcRn inhibitors. The AE reports of these five drugs as PS and with indications for MG since their FDA approval were retrieved using the generic name and brand name respectively. The cases specifically indicated in the INDI_PT field that contained terms related to MG were retained. **Figure 1** provides a detailed process overview.

These AEs were standardized and classified using the preferred terms (PTs) and SOCs from the regulatory activity medical dictionary (MedDRA, version 28.1) to improve the reliability of statistical analysis (21).

### 2.3 Statistical analysis

In this pharmacovigilance study, we employed disproportionality analysis to identify potential associations between drugs and AEs. Three distinct disproportionality analysis methods were utilized to evaluate AE signals, including the ROR method (22, 23), the MHRA method (24), and the BCPNN method (25). All three methods performed statistical analyses based on 2×2 contingency tables (as shown in **Supplementary Table 1**), with their respective calculation formulas and signal detection criteria detailed in **Supplementary Table 2**. For this study, we focused on AEs that were concurrently identified as risk signals by all three algorithms, where higher signal values indicated stronger associations between drugs and AEs. All statistical analyses were conducted using R4.4.3 software.

### 2.4 Network toxicological analysis

Network pharmacology can rapidly predict the target sites and pathways of diseases. In this study, a protein interaction network of complement C5 and FcRn was constructed using the STRING database, and potential target groups for complement C5 inhibitors and FcRn inhibitors were screened out. Key targets related to SOCs (26, 27) were collected through GeneCards. The Venn diagram was used to identify the intersection of potential drug targets and SOC-related targets, which are potential toxic targets of drug-related SOCs. The protein-protein interaction (PPI) network map of potential toxic targets was constructed using the STRING database and Cytoscape 3.7.2 software to determine the key targets (28). The DAVID database was used to perform Gene Ontology (GO) and Kyoto Encyclopedia of Genes and Genomes (KEGG) enrichment analysis on the potential toxic targets of key SOCs.

## 3 Results

### 3.1 Basic characteristics of AEs and population

**Figure 2:**
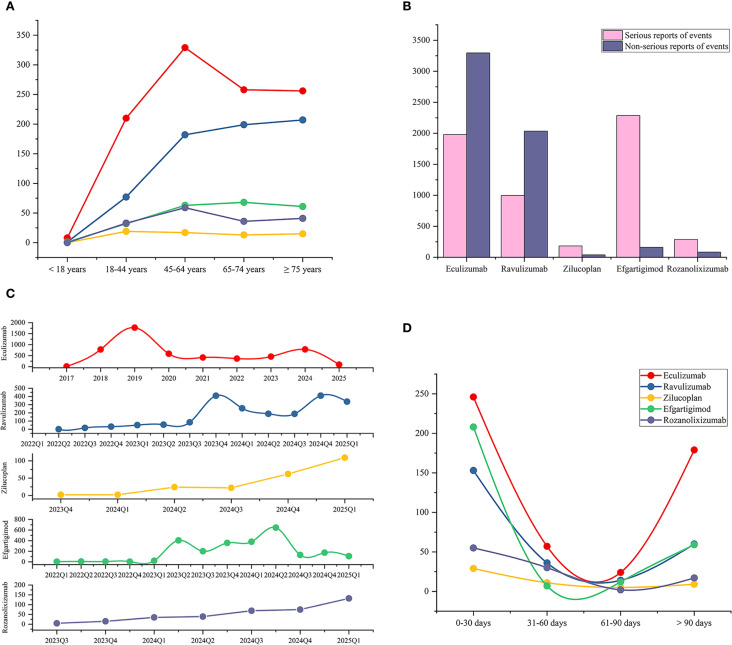
Epidemiological characteristics of adverse events associated with complement C5 inhibitors and FcRn inhibitors, including patient age distribution, reporting year, serious outcomes, and time-to-onset analysis. **(A)** Age distribution of patients with AE reports associated with the five drugs. **(B)** The proportion of serious outcomes in the AE reports for each drug. **(C)** Number of AE reports per quarter for each drug (annual for eculizumab). **(D)** Onset time of five drugs associated adverse events (Statistical analyses were performed only on reports that provided the onset time).

**Table 1** describes the demographic and event characteristics of AE reports for complement C5 inhibitors and FcRn inhibitors used in treating MG. Except for ravulizumab, where the number of female reporters (969, 31.97%) was slightly lower than that of males (1094, 36.09%), all other drugs showed a higher proportion of female reporters than male reporters. Among the population reporting adverse reactions, the average age of AE reporters across all five drugs was around 60 years (**Figure 2A**), a characteristic that may be related to the age distribution of MG onset (29). It is worth noting that the indication of eculizumab is for gMG in adult and pediatric patients aged 6 years and above who are anti-AChR antibody positive. However, the reported patient ages include those younger than 1 year old, which should be considered as off-label use. In their reports, there is indeed an entry for off-label use of this drug as an AE. Consumers accounted for the highest proportion of AE reporters (55.46%-73.75%), reflecting increasing public vigilance regarding medication safety. According to the FAERS database, the United States contributed the highest number of AE reports (79.64%-92.55%), this might be related to the source of the database. The most frequently recorded severe outcomes were hospitalization (13.03%-52.49%), followed by death, life-threatening and disability. The proportions of serious reports of events were 37.53% for eculizumab and 32.89% for ravulizumab, while obviously higher rates were observed with zilucoplan (82.35%), efgartigimod (93.46%), and rozanolixizumab (77.57%). This discrepancy warrants further investigation.

**Table 1:** Demographic and clinical characteristics of patients with complement C5 inhibitors and FcRn inhibitors associated adverse events reported in the FAERS database.

Characteristes	Case number				
	C5 complement inhibitor			FcRn inhibitor	
	Eculizumab	Ravulizumab	Zilucoplan	Efgartigimod	Rozanolixizumab
Gender					
Female (%)	2520 (47.76)	969 (31.97)	123 (55.66)	211 (8.63)	214 (57.84)
Male (%)	2149 (40.73)	1094 (36.09)	93 (42.08)	187 (7.65)	145 (39.19)
Not Specifed (%)	607 (11.50)	968 (31.94)	5 (2.26)	2048 (83.73)	11 (2.97)
Age (years)					
< 18 (%)	8 (0.15)	1 (0.03)	0 (0.00)	1 (0.04)	0 (0.00)
18-44 (%)	210 (3.98)	77 (2.54)	19 (8.60)	32 (1.31)	33 (8.92)
45-64 (%)	329 (6.24)	182 (6.00)	17 (7.69)	63 (2.58)	59 (15.95)
65-74 (%)	258 (4.89)	199 (6.57)	13 (5.88)	68 (2.78)	36 (9.73)
≥75 (%)	256 (4.85)	207 (6.83)	15 (6.79)	61 (2.49)	41 (11.08)
Not Specifed (%)	4215 (79.89)	2365 (78.03)	157 (71.04)	2221 (90.80)	201 (54.32)
Mean (SD)	60.16 (17.63)	65.26 (15.33)	58.03 (19.01)	64.45 (16.97)	60.83 (17.44)
Median (Q1, Q3)	64 (48,74)	69 (58,76)	60 (43,74)	67 (53,76)	62 (51,74)
Min, Max	1.94	14.97	21.89	17.93	19.99
Body weight (kg)					
N (Missing)	367 (4909)	259 (2772)	159 (62)	191 (2255)	85 (285)
Mean (SD)	94.31 (37.10)	86.55 (29.69)	91.60 (28.80)	84.54 (29.90)	85.72 (33.53)
Median (Q1. Q3)	86.20 (69.00.111.06)	80.90 (63.49, 108.42)	85.28 (72.65,107.13)	84.80 (69.85,102.50)	81.65 (65.00,98.88)
Min. Max	30.80,290.00	28.00.208.65	32.70,214.00	33.65.220.50	27.00.263.00
Reporter					
Consumer (%)	4273 (80.99)	1681 (55.46)	138 (62.44)	1804 (73.75)	219 (59.19)
Pharmacist (%)	82 (1.55)	46 (1.52)	8 (3.62)	66 (2.70)	21 (5.68)
Physician (%)	476 (9.02)	514 (16.96)	34 (15.38)	312 (12.76)	39 (10.54)
Lawyer (%)	0 (0.00)	0 (0.00)	1 (0.45)	0 (0.00)	0 (0.00)
Healthcare Professional (%)	262 (4.97)	178 (5.87)	40 (18.10)	250 (10.22)	91 (24.59)
Not Specifed (%)	183 (3.47)	612(20.19)	0 (0.00)	14(0.57)	0 (0.00)
Reported countries (Top3)					
United States of America (%)	4883 (92.55)	2805 (92.54)	176 (79.64)	2013 (82.30)	325 (87.84)
Japan (%)	151 (2.86)	(3.00)	19 (8.60)	221 (9.04)	26 (7.03)
Germany (%)	61 (1.16)			44(1.80)	11 (2.97)
Canada (%)		29 (0.96)			
France (%)			14 (6.33)		
Serious criteria					
Serious reports of events (%)	1980 (37.53)	997 (32.89)	182 (82.35)	(93.46)	287 (77.57)
Non-serious reports of events (%)	3296 (62.47)	2034 (67.11)	39 (17.65)	160 (6.54)	83 (22.43)
Serious outcome					
Life-Threatening (%)	42 (0.79)	18 (0.59)	15 (6.79)	170 (6.95)	5 (1.35)
Hospitalization Initial or Prolonged (%)	986 (18.69)	395 (13.03)	76 (34.39)	1284 (52.49)	120 (32.43)
Disability (%)	8 (0.15)	5 (0.16)	1 (0.45)	8 (0.33)	2 (0.54)
Death (%)	191 (3.62)	87 (2.87)	6 (2.71)	227 (9.28)	5 (1.35)
Congenital Anomaly (%)	1 (0.02)	0 (0.00)	0 (0.00)	0 (0.00)	0 (0.00)
Required Intervention to Prevent Permanent Impairment/Damage (%)	0 (0.00)	2(0.07)	0 (0.00)	8 (0.33)	1 (0.27)
Other Serious (%)	1626 (30.82)	887 (29.26)	151 (68.33)	1242 (50.78)	241 (65.14)
Onset time (days)					
0-30 (%)	246 (4.66)	153 (5.05)	29 (13.12)	208 (8.50)	55 (14.86)
31-60 (%)	57 (1.08)	36 (1.19)	11 (4.98)	7 (0.29)	30 (8.11)
61-90 (%)	24 (0.45)	14 (0.46)	5 (2.26)	12 (0.49)	2 (0.54)
> 90 (%)	179 (3.39)	60 (1.98)	9 (4.07)	59 (2.41)	17 (4.59)
Not Specifed (%)	4770 (90.41)	2768 (91.32)	167(75.57)	2160 (88.31)	266 (71.89)
Mean (SD)	206.57 (552.40)	69.44 (138.63)	45.22 (52.51)	69.44 (116.59)	83.08 (184.56)
Median (Q1, Q3)	35 (19,175)	14 (1.71)	23(7,61)	22 (21.60)	30 (13.42)
Min, Max	1,9636	1,1271	1,202	1,690	1,1206

FAERS, FDA Adverse Event Reporting System.

Analysis of pharmacovigilance data showed that, except for eculizumab, the number of AE reports for other drugs used in MG treatment exhibited some quarterly fluctuations but generally increased year by year. For eculizumab, after its approval for MG treatment in 2017, the number of reports surged in the following two years—2018 (780, 14.79%) and 2019 (1773, 33.61%)—likely due to initial clinical experience accumulation, before stabilizing gradually from 2020 onward (**Figure 2C**).

Regarding the distribution of AE onset time (**Figure 2D**), AEs for all five drugs were more likely to manifest early, with the highest number of reports occurring within 0–30 day. Due to the lack of many reports on the onset time of AEs, we calculated the median onset time, which is a more representative indicator reflecting the overall data distribution. We found that the median onset time was within 35 days (**Table 1**). The reports with AEs onset time exceeding 90 days are the second most numerous, this highlights the need for continuous monitoring of potential delayed-onset AEs associated with these drugs. We further mapped all the reports that provided the onset time to the corresponding PTs through the primaryID (**Supplementary Table 8**). Among the PTs that showed signals for eculizumab, arthralgia, gastric cancer, pyelonephritis mostly occurred within 30 days, while memory impairment mostly occurred after 90 days. Among the reported AEs of ravulizumab, back pain, abnormal sensations, arthritis mostly occurred within 30 days, while photophobia, speech disorder, gait disturbance mostly occurred after 90 days. In the PTs with signals in zilucoplan, injection site pain persists throughout the treatment period. Viral infections occur mostly in the early stage (within the first 30 days). Injection site erythema, induration and pruritus usually occur after 90 days. For efgartigimod, AEs such as respiratory syncytial virus infection, blood blister, dyslalia mostly occur in the early stage of treatment (0–30 days), while AEs such as congestive heart failure, catarrh, pulmonary congestion mostly occur later (after 90 days). For rozanolixizumab, conditions such as herpes zoster, rash, and aseptic meningitis occur within 30 days, while influenza-like illness occurs after 90 days.

### 3.2 Distribution of AE frequency at the PT level

**Figure 3:**
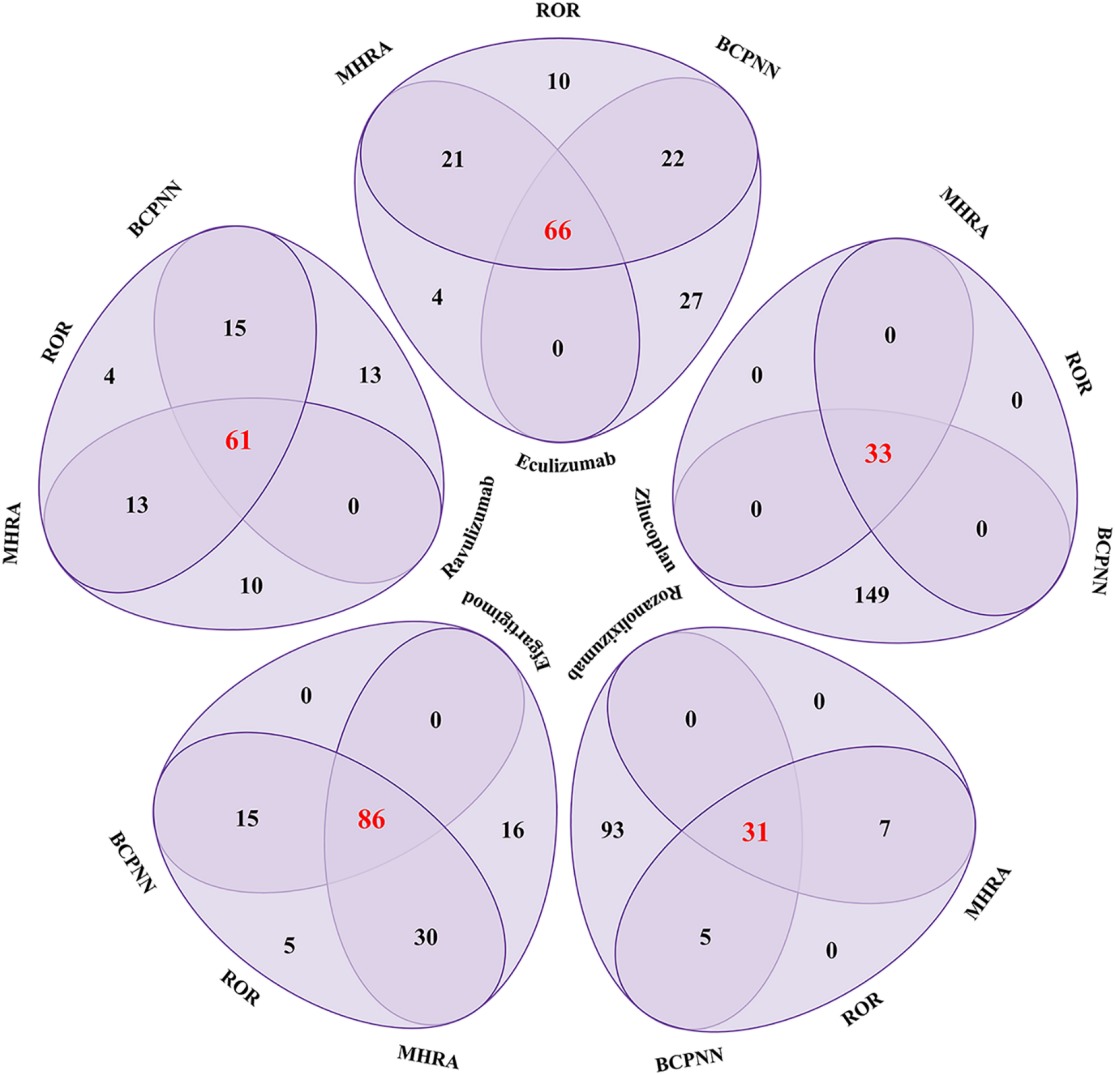
Venn diagram analysis results of complement C5 inhibitors and FcRn inhibitors for positive PT signal values. ROR, Reporting Odds Ratio; MHRA, the UK Medicines and Healthcare products Regulatory Agency; BCPNN, the Bayesian Confidence Propagation Neural Network.

As shown in **Figure 3**, among PTs with positive signals by all three statistical methods, the number of PT species of complement C5 inhibitors was eculizumab (66), ravulizumab (61), zilucoplan (30) respectively. The number of PT species of FcRn inhibitors was efgartigimod (86) and rozanolixizumab (29). Among them, zilucoplan and rozanolixizumab reported fewer PT species, which may be related to their shorter time on the market.

**Figure 4:**
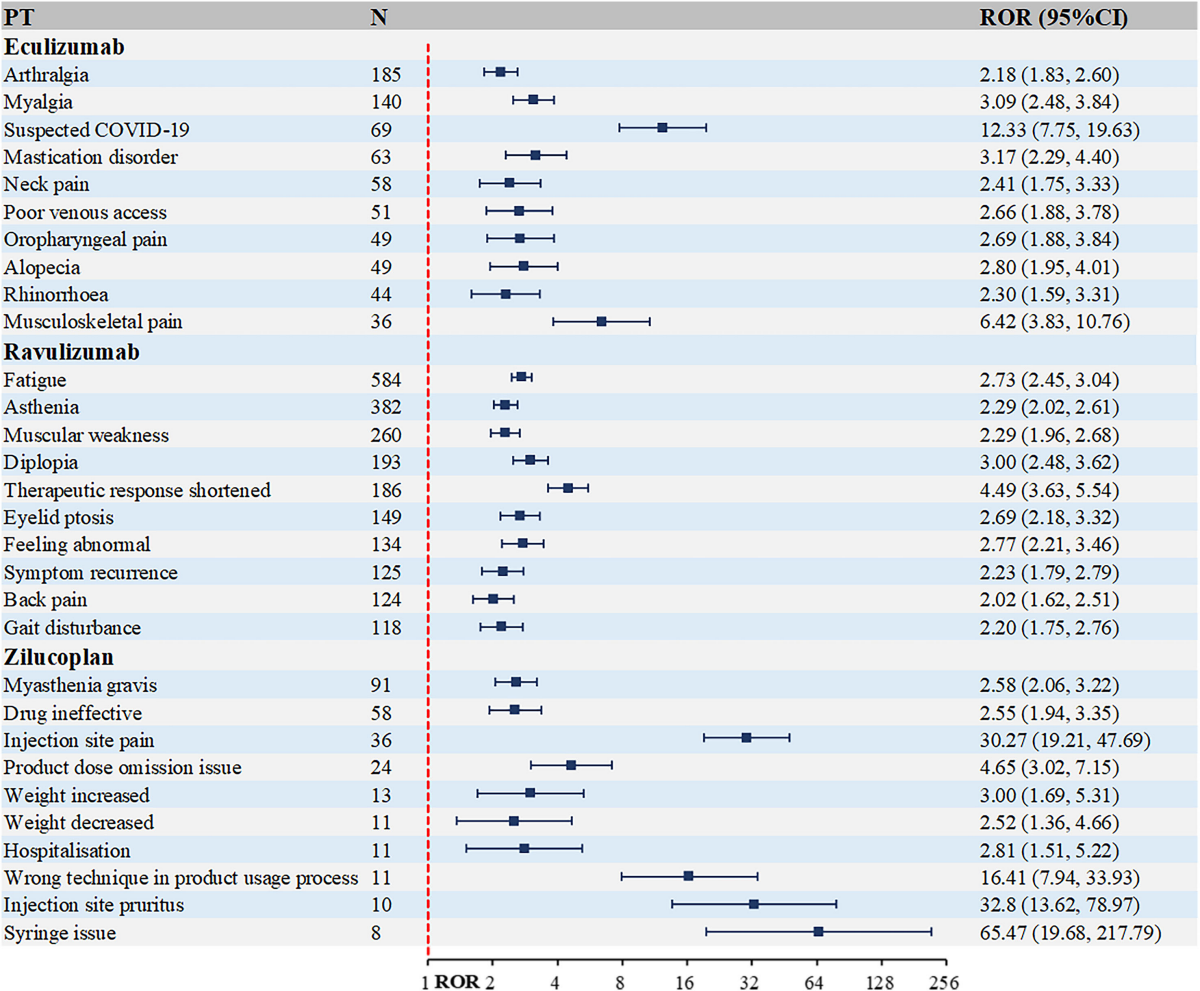
Top 10 PTs ranked by the number of reports for complement C5 inhibitors. PT, preferred term; ROR, Reporting Odds Ratio.

**Figure 5:**
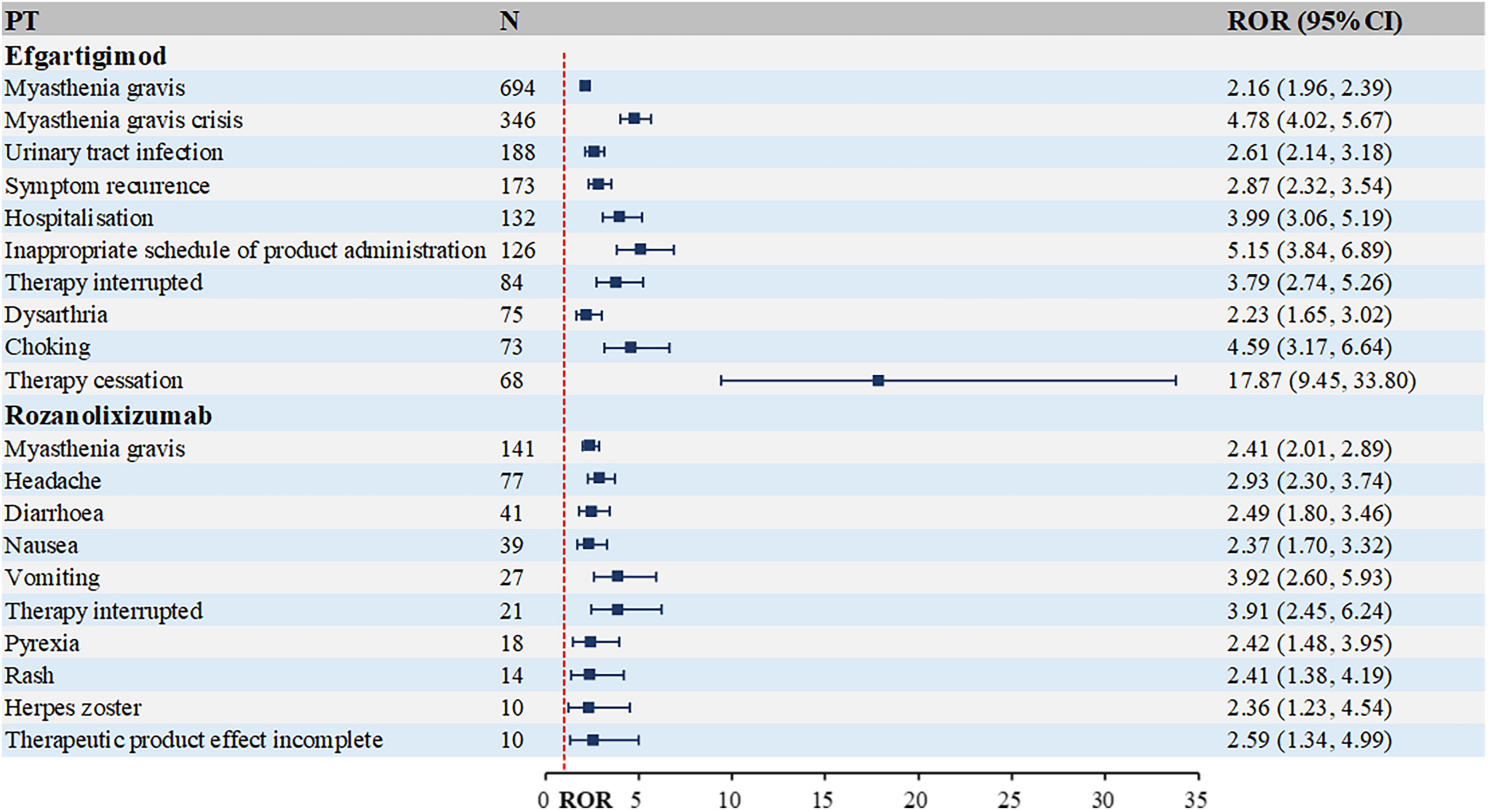
Top 10 PTs ranked by the number of reports for FcRn inhibitors. PT, preferred term; ROR, Reporting Odds Ratio.

The top 10 most frequently occurring PTs associated with complement C5 inhibitors and FcRn inhibitors in MG treatment are presented in **Figures 4** and **5**. **Figure 4** shows that the highest number of reports for eculizumab was arthralgia [n=185, ROR (95% Cl)=2.18 (1.83, 2.6)]. Other musculoskeletal and connective tissue disorders were also reported, such as myalgia, mastication disorder and neck pain. While for ravulizumab, the highest number of reports was fatigue [n=584, ROR (95% Cl)=2.73 (2.45, 3.04)]. Among the top 10 PTs, there were 6 systemic manifestations, including fatigue, asthenia, therapeutic response shortened, feeling abnormal, symptom recurrence and gait disturbance. As a new small molecule cyclic peptide complement C5 inhibitor, zilucoplan showed weight increased and weight decreased, which were not mentioned in the instructions.

As evident from **Figure 5**, FcRn inhibitors demonstrate a higher infection risk profile compared to complement C5 inhibitors. Notable examples include: efgartigimod: urinary tract infection [n=188, ROR (95% CI)=2.61 (2.14-3.18)], rozanolixizumab: herpes zoster [n=10, ROR (95% CI)=2.36 (1.23-4.54)]. Additionally, rozanolixizumab reports showed a higher frequency of gastrointestinal disorders, particularly diarrhea, nausea and vomiting.

### 3.3 Distribution of AE Signal Strength at the PT level

The top 10 PTs ranked by ROR signal strength for these five therapeutic agents in MG management are displayed in **Table 2** (complement C5 inhibitors) and **Table 3** (FcRn inhibitors). For eculizumab, the highest ROR was observed for Inappropriate schedule of drug administration [n=13, ROR (95% CI)=27.78 (6.27-123.1)], followed by General symptom [n=6, ROR (95% CI)=25.63 (3.09-212.88)]. It is noteworthy that gastric cancer and embolic stroke, ranked fourth and ninth in ROR signal intensity respectively, are previously unreported serious adverse reactions. Ravulizumab showed the strongest signal for Paranasal sinus hypersecretion [n=4, ROR (95% CI)=15.39 (1.72-137.74)]. In addition, there are psoriatic arthropathy, peripheral vascular disorders, which are not mentioned in the label. Zilucoplan demonstrated predominantly high ROR values for injection site physical injuries, likely associated with its subcutaneous administration route. Efgartigimod exhibited the highest RORs for: dyspnoea at rest [n=27, ROR (95% CI)=25.94 (7.87-85.52)], pulmonary congestion [n=9, ROR (95% CI)=25.91 (3.28-204.5)]. Rozanolixizumab showed peak ROR values for: infusion site pruritus [n=4, ROR (95% CI)=17.15 (4.60-63.94)], meningitis aseptic [n=3, ROR (95% CI)=10.73 (3.66-31.41)].

**Table 2:** Top 10 PTs ranked by ROR signal strength for complement C5 inhibitors.

Complement C5 inhibitors	PT	SOC	Case number	ROR (95%CI)	PRR	x2	IC025
E culizumab	Inappropriate schedule of drug administration	Injury, poisoning and procedural complications	13	27.78 (6.27,123.10)	27.75	44.71	1.06
	General symptom	General disorders and administration site conditions	6	25.63 (3.09,212.88)	25.62	20.28	0.69
	Multimorbidity		4	17.08 (1.91,152.85)	17.08	12.11	0.42
	Gastric cancer	Neoplasms benign, malignant and unspecified	5	21.35 (2.49,182.80)	21.35	16.16	0.57
	Escherichia sepsis	Infections and infestations	4	17.08 (1.91,152.85)	17.08	12.11	0.42
	Axillary mass	Musculoskeletal and connective tissue disorders	3	12.81 (1.33,123.17)	12.81	8.17	0.21
	Blood creatin ine abnormal	Investigations	3	12.81 (1.33,123.17)	12.81	8.17	0.21
	Red cell distribution width increased		3	12.81 (1.33,123.17)	12.81	8.17	0.21
	Embolic stroke	Nervous system disorders	3	12.81 (1.33,123.17)	12.81	8.17	0.21
	Eye symptom	Eye disorders	3	12.81 (1.33,123.17)	12.81	8.17	0.21
Ravulizumab	Paranasal sinus hypersec retion	Respiratory, thoracic and mediastinal disorders	4	15.39 (1.72,137.74)	15.39	10.76	0.32
	Nocturnal dyspnoea		3	11.54 (1.20,110.99)	11.54	7.22	0.11
	Upper respiratory tract congestion		3	11.54 (1.20,110.99)	11.54	7.22	0.11
	Herpes simplex	Infections and infestations	3	11.54 (1.20,110.99)	11.54	7.22	0.11
	Meningococcal infection		5	9.62 (1.87,49.60)	9.62	11.03	0.30
	International normalised ratio increased	Investigations	3	11.54 (1.20,110.99)	11.54	7.22	0.11
	Peripheral vascular disorder	Vascular disorders	3	11.54 (1.20,110.99)	11.54	7.22	0.11
	Intervertebral disc disorder		3	11.54 (1.20,110.99)	11.54	7.22	0.11
	Psoriatic arthropathy		3	11.54 (1.20,110.99)	11.54	7.22	0.11
	Trigger finger		3	11.54 (1.20,110.99)	11.54	7.22	0.11
Zilucoplan	Syringe issue	Product issues	8	65.47 (19.68,217.79)	64.97	168.02	2.68
	Needle issue		6	49.01 (13.81,173.94)	48.73	112.23	2.46
	Injection site mass	General disorders and administration site conditions	4	130.45 (14.57,1168.18)	129.94	102.37	2.72
	Injection site induration		3	97.74 (10.16,940.47)	97.46	71.61	2.56
	Injection site irritation		3	48.87 (8.16,292.79)	48.73	56.11	2.25
	Injection site pruritus		10	32.80 (13.62,78.97)	32.485	152.67	2.406
	Injection site pain		36	30.27 (19.21, 47.69)	29.237	517.862	2.857
	Injection site bruising		7	22.89 (8.70.60.26)	22.74	85.622	2.045
	Wrong technique in product usage process	Injury, poisoning and procedural complications	11	16.41 (7.94,33.93)	16.243	104.999	1.979
	Knee operation	Surgical and medical procedures	3	16.29 (4.07,65.22)	16.243	28.616	1.483

PT, preferred terms; SOC, system organ categories; ROR, Reporting odds ratio; PRR, Proportional reporting ratio; IC, Information component; IC025, The lower limit of the 95%CI of IC.

**Table 3:** Top 10 PTs ranked by ROR signal strength for FcRn inhibitors.

FcRn inhibitors	PT	SOC	Case number	ROR (95% CI)	PRR	%2	IC025
E fgartigimod	Dyspnoea atrest	Respiratory, thoracic and mediastinal disorders	27	25.94 (7.87,85.52)	25.89	64.64	1.04
	Pulmonary congestion		9	25.91 (3.28,204.50)	25.89	21.54	0.60
	Loss of therapeutic response	General disorders and administration site conditions	5	14.39(1.68,123.16)	14.38	10.38	0.23
	Therapeutic product ineffective		15	21.60 4.94,94.46)	21.57	34.63	0.80
	Thymectomy	Surgical and medical procedures	24	13.83 (5.28,36.26)	13.81	49.18	0.90
	Stent placement		7	20.15 (2.48,163.76)	20.13	15.91	0.45
	Therapy cessation		68	17.87 (9.45,33.8)	17.78	150.20	1.25
	Tooth extraction		6	17.27 (2.08,143.43)	17.26	13.13	0.35
	Immobile	Social circumstances	6	17.27 (2.08,143.43)	17.26	13.13	0.35
	Lumbar vertebral fracture	Injury, poisoning and procedural complic ations	6	17.27 (2.08,143.43)	17.26	13.13	0.35
Rozanolixizumab	Infusion site pruritus	General disorders and administration site conditions	4	17.15 (4.60,63.94)	17.12	33.73	1.44
	Injection site bruising		3	4.29 (1.24,14.81)	4.28	6.29	0.14
	Injection site swelling		6	5.60 (2.28,13.76)	5.58	17.91	0.72
	Adverse event		5	8.94 (3.15,25.40)	8.92	24.81	1.04
	Injection site erythema		8	5.05( 2.34,10.93)	5.03	20.96	0.77
	Meningitis aseptic	Infections and infestations	5	10.73 (3.66,31.41)	10.70	29.32	1.19
	Plasmapheresis	Surgical and medical procedures	4	5.72 (1.90,17.24)	5.71	12.26	0.55
	Taste disorder	Nervous systemdisorders	3	5.36 (1.51,19.00)	5.35	8.49	0.36
	Wrong technique in product usage process	Injury, poisoning and procedural complications	6	4.60 (1.90,11.12)	4.59	13.86	0.54
	Eating disorder	Psychiatric disorders	4	4.29 (1.46,12.56)	4.28	8.38	0.28

PT, preferred terms; SOC, system organ categories; ROR, Reporting odds ratio; PRR, Proportional reporting ratio; IC, Information component; IC025, The lower limit of the 95%CI of IC.

### 3.4 PT analysis in subgroups

In the gender subgroup (**Supplementary Figure 1**), females treated with eculizumab were more likely to report fatigue and pain, while males more frequently reported arthralgia and myalgia. Among those treated with ravulizumab, females more commonly reported fatigue and muscular weakness, whereas males reported fatigue and asthenia more often. For zilucoplan, the top2 AEs reported by males were myasthenia gravis and injection site pain, while for females, they were myasthenia gravis and drug ineffective. Regarding FcRn inhibitors (**Supplementary Figure 2**), the number of reports for the PT “Death” associated with efgartigimod is high in both males (31) and females (22). With rozanolixizumab, males more frequently reported myasthenia gravis and headache, while females reported speech disorder and therapy interruption more often.

Among the reported population (**Supplementary Figure 3**), consumers treated with eculizumab more commonly reported myalgia and pyrexia, whereas health professional more frequently reported myasthenia gravis and suspected COVID-19. For ravulizumab, consumers reported fatigue and asthenia more often, while health professional reported symptom recurrence and shortened therapeutic response more frequently. Regarding zilucoplan, the top2 AEs reported by consumers were myasthenia gravis and drug ineffective, whereas health professional reported product dose omission issue and weight decrease more often. For efgartigimod (**Supplementary Figure 4**), consumers more frequently reported myasthenia gravis and myasthenia gravis crisis, while health professional were more aware of myasthenia gravis crisis and symptom recurrence. With rozanolixizumab, consumers are often acutely aware of their own somnolence and neck pain more often, while health professional reported headache and eyelid ptosis more frequently.

### 3.5 SOC associated with positive AE signals

**Figure 6:**
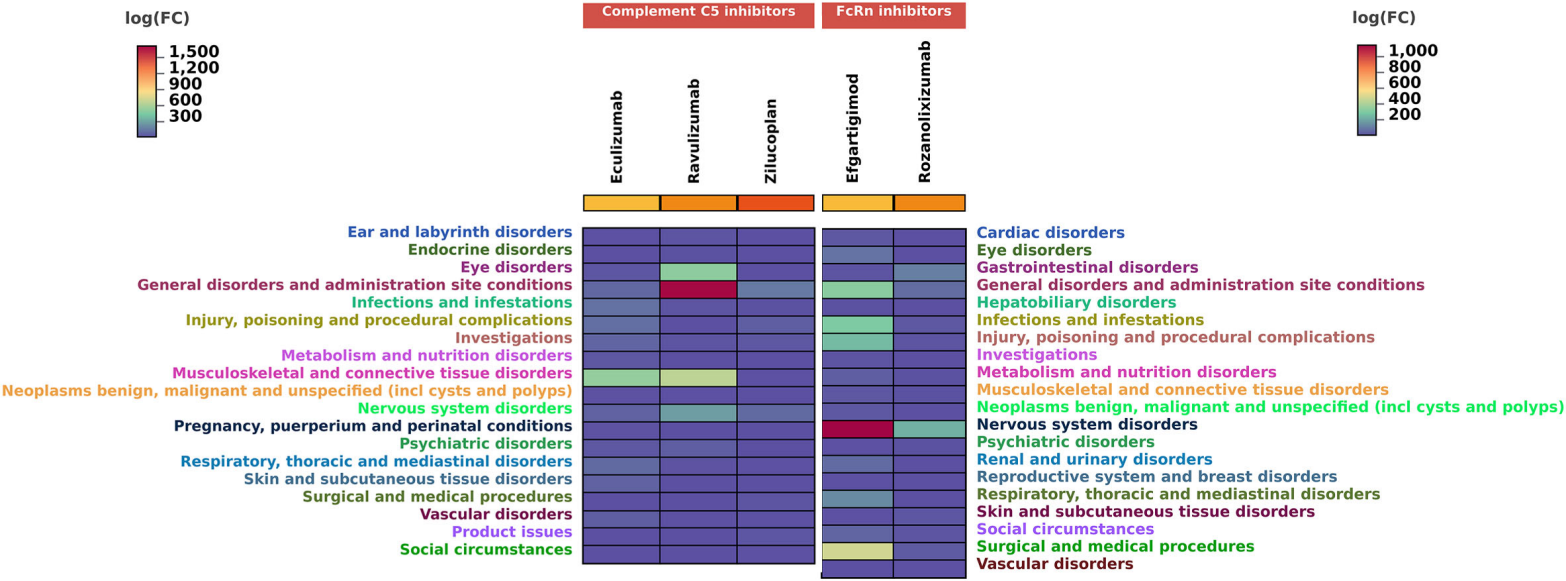
Number of reports contained in SOCs involved in new potential ADE signals. SOC, system organ categories.

To investigate the SOC distribution of adverse drug reactions associated with two novel classes of biologics in MG treatment, we have calculated the corresponding SOCs for the identified positive-signal PTs according to the MedDRA hierarchy. As shown in **Figure 6**, complement C5 inhibitors involved 19 SOCs, with the majority of reported PTs falling under general disorders and administration site conditions, followed by musculoskeletal and connective tissue disorders. FcRn inhibitors covered 20 SOCs, where most reported PTs belonged to nervous system disorders, with general disorders and administration site conditions being the second most frequent.

### 3.6 GO and KEGG enrichment analysis of potential toxic targets of key SOCs

**Figure 7:**
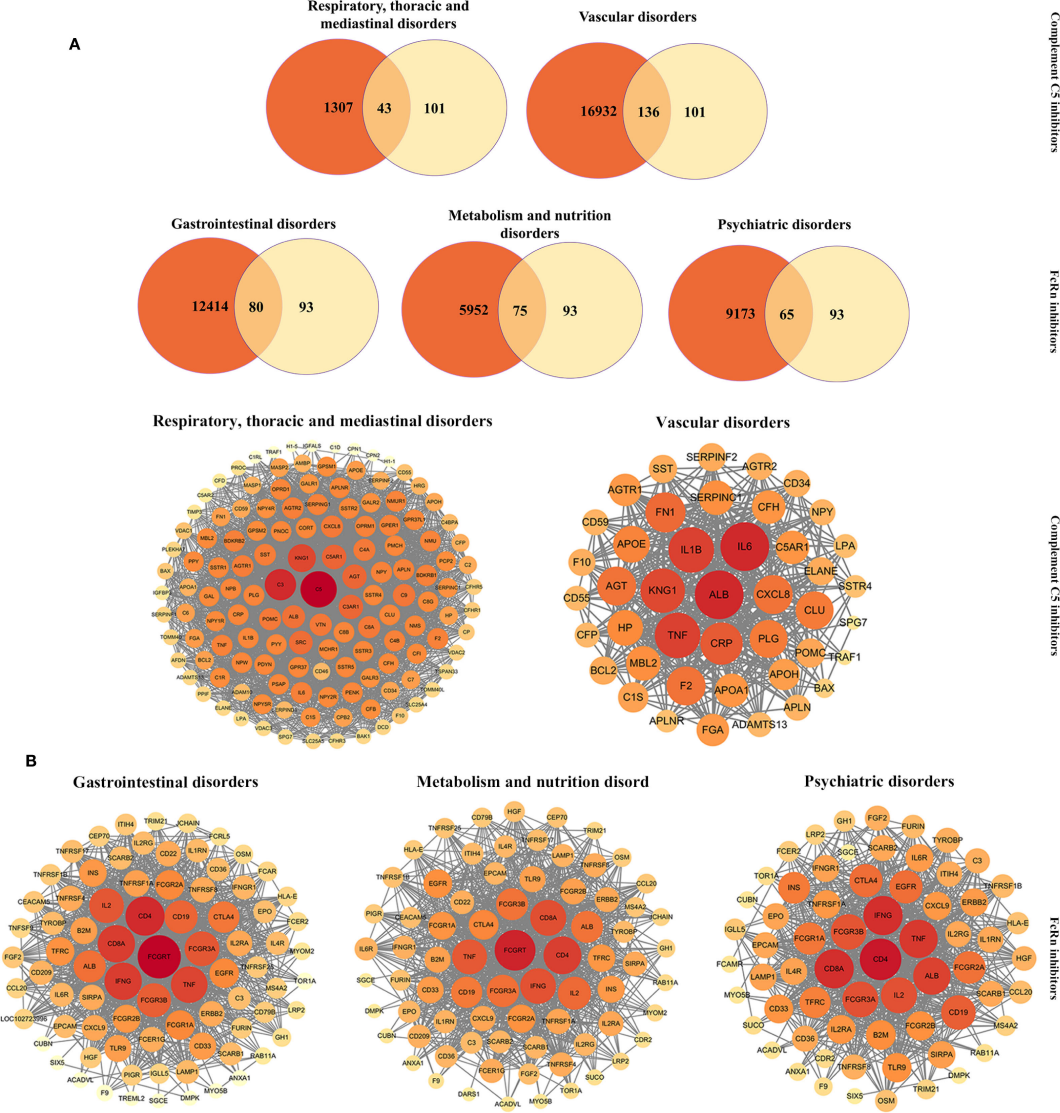
PPI networks of potential toxictargets of complement C5 inhibitors and FcRn inhibitors. **(A)**Venn diagram of prediction targets and key SOCs of complement C5 inhibitors and FcRn inhibitors. **(B)** PPI network map of common targets for complement C5 inhibitors (and two key SOCs) and FcRn inhibitors (and three key SOCs). PPI, protein-protein interaction; SOC, system organ categories.

To further investigate the toxicological mechanisms of complement C5 inhibitors and FcRn inhibitors, we selected the SOCs of interest and identified their associated potential toxicity targets through PPI analysis (**Figure 7A**). The key nodes for complement C5 inhibitors mainly included C5, C3, KNG1, ALB and IL6, while nodes such as FCGRT, IL2, IFNG, CD4 and TNF significantly contributed to the toxicity of FcRn inhibitors.

**Figure 8:**
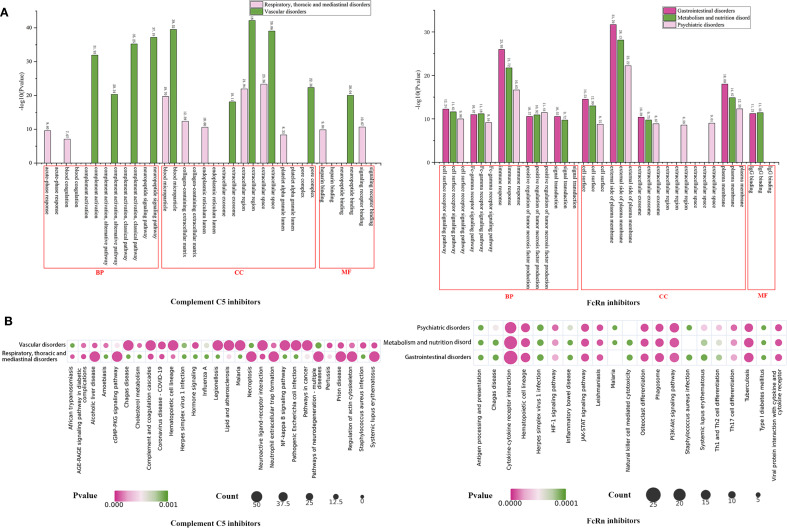
GO and KEGG enrichment analysis of potential toxictargets of key SOCs of complement C5 inhibitors and FcRn inhibitors. GO functional **(A)** and KEGG pathway **(B)** enrichment analysis of potential toxictargets of key SOCs. GO, Gene Ontology; KEGG, Kyoto Encyclopedia of Genes and Genomes; SOC, system organ categories.

We further conducted GO (**Figure 7B**) and KEGG (**Figure 8**) enrichment analyses using the DAVID database. In the GO enrichment analysis of the two key SOC toxicity targets for complement C5 inhibitors, the top 10 GO terms included 6 biological processes, 8 cellular components and 3 molecular functions. For the GO enrichment analysis of the three key SOC toxicity targets for FcRn inhibitors, the top 10 GO terms comprised, 5 biological processes, 6 cellular components and 1 molecular function. In the KEGG enrichment analysis of the toxicity targets, the top 20 significant pathways for complement C5 inhibitors primarily included diabetic complications, complement and coagulation cascades, COVID-19 and the AGE-RAGE signaling pathway in systemic lupus erythematosus. Similarly, the top 20 significant pathways for FcRn inhibitors mainly involved cytokine-cytokine receptor interaction, hematopoietic cell lineage, the PI3K-Akt signaling pathway and tuberculosis.

## 4 Discussion

In recent years, the use of biologics for treating MG has increased significantly. To standardize the clinical application of these drugs, research on their AEs has become crucial. This study analyzed AEs associated with complement C5 inhibitors and FcRn inhibitors recorded in the FAERS database following their approval and market introduction for MG treatment. In addition to confirming previously documented adverse reactions in prescribing information as well as comorbidities of MG such as thyroid disorders, systemic lupus erythematosus, and vitiligo (32), our research also identified additional AEs.

### 4.1 Overall distribution of AEs

Eculizumab was approved by the FDA for MG a decade following its initial market entry. Commonly associated AEs include nasopharyngitis, as well as more serious but well-established risks such as meningococcal bacteremia and meningococcal septicemia, which have been extensively documented in the literature (30, 33, 34). Additionally, we identified two previously unlabeled AEs of concern: gastric cancer and embolic stroke, which did not occur when eculizumab was used for other indications (such as neuromyelitis optica spectrum disorders (NMOSD), paroxysmal nocturnal hemoglobinuria (PNH) and atypical hemolytic uremic syndrome (aHUS)). Ravulizumab was associated with several unlisted AEs, including psoriatic arthropathy, hypoacusis and peripheral vascular disorders. However, ravulizumab has not been reported to cause above AEs when used to treat PNH. Zilucoplan, administered via subcutaneous injection, demonstrated a higher frequency of injection site-related AEs, likely due to its route of administration. In a trial involving 174 MG patients, ecchymosis at the injection site was the most common treatment-related AE, occurring in 16% (n=14) (35). Notably, it also showed a strong correlation with weight fluctuations (gain or loss), an unreported finding warranting heightened vigilance. Due to the relatively short period since these drugs were launched on the market and the related safety studies are quite limited, these new suspicious signals discovered in this study still lack direct literature support.

Efgartigimod demonstrated significant hepatorenal toxicity signals, including hepatic AEs (Metastases to liver, hepatic failure) and renal disorders (Nephrolithiasis, dysuria) (**Supplementary Table 6**). Yang et al.’s analysis of FAERS data (2022 Q1–2024 Q2) identified a strong unexpected signal for nephrolithiasis (ROR 8.13, PRR 7.99, IC 2.99, EBGM 7.95) (36). Notably, we detected previously unreported safety concerns male reproductive system toxicity (Prostatitis, prostate cancer) and cardiovascular events (Angina pectoris, congestive cardiac failure) (**Supplementary Table 6**). Yu et al. also identified new and significant adverse reaction signals such as sepsis, atrial fibrillation, and transient ischemic attack in their study of FAERS data from 2022 Q1 to 2023 Q4 (37). These findings represent novel safety signals requiring further investigation. Rozanolixizumab showed more gastrointestinal disorders, including the already labeled diarrhea and nausea, as well as unlabeled vomiting and dyspepsia (**Supplementary Table 7**). In a randomized, double-blind, placebo-controlled, adaptive phase 3 study of rozanolixizumab involving 200 participants, Bril V et al. reported that the most common AEs included headache, diarrhea and fever (31). Cooper N et al. reported in two randomized, double-blind, placebo-controlled phase 3 studies and their open-label extension studies of rozanolixizumab that the most common treatment-related AEs included headache, fever and nausea (38). By correlating these PTs with their onset time, we found that gastric cancer (eculizumab), liver metastasis, and prostate cancer (efgartigimod) had occurred within 30 days (**Supplementary Table 9**). Therefore, these AEs should be identified as false positive signals. Our analysis indicates no causal relationship between these events and the drugs.

### 4.2 Information from subgroup

Gender-based analysis showed that females more frequently reported less severe AEs such as poor quality sleep, dry skin, migraine and alopecia. In contrast, males tended to report more serious AEs, such as sepsis associated with eculizumab [n=27, ROR (95% CI)=2.20 (1.34, 3.62)] and deep vein thrombosis for zilucoplan [n=3, ROR (95% CI)=6.12 (1.65, 22.67)]. Additionally, the number of reported deaths associated with efgartigimod was higher in males (31) than in females (22). These AEs may be attributed to a greater reduction in the immune system’s defenses in males following the drug, impairing their ability to fight off new infections. Additionally, males may exhibit a greater tendency to delay seeking medical care, leading to conditions being identified at a more severe stage.

Reporter analysis indicated that consumers more commonly reported subjective experiences and impacts on quality of life, such as alopecia, fatigue, asthenia, drug ineffectiveness, somnolence and pruritus etc. In comparison, healthcare professionals focused more on objective clinical findings, including hospitalization, deep vein thrombosis, suspected COVID-19 and nasopharyngitis etc. These differences contribute to a comprehensive understanding of medication safety from multiple perspectives.

### 4.3 Fatal events related to two novel classes of biologics

In terms of fatal PTs, we observed that death was the most frequently reported outcome for all five drugs (**Supplementary Table 9**). In addition, several other PTs of eculizumab such as pneumonia (9/394), suspected COVID-19 (8/394) and dyspnoea (6/394) were also reported. ravulizumab also reported cardiac arrest (3/136) and myocardial infarction (3/136). Pneumonia (17/774) and urinary tract infection (16/774) were also reported for efgartigimod. Zilucoplan and rozanolixizumab have relatively few reported cases of death due to their relatively short time on the market.

These fatal reactions are typically compounded by its adverse effects. When patients have a lower resistance or the medication is used improperly, they may be more susceptible to infections, such as pneumonia, urinary tract infection and COVID-19 infection. In more severe cases, it may even cause cardiac arrest and myocardial infarction.

Similar to the discrepancy in the number of reported serious events and serious outcomes among the five drugs, there is also variation in the reported number of fatal PTs. These findings do not appear to align with data from clinical trials or real-world evidence to date. Niklas Huntemann et al. reported comparable efficacy in a study of 153 MG patients from 8 German specialized MG centers receiving either complement C5 inhibitors (26 on eculizumab, 80 on ravulizumab) or FcRn inhibitor (47 on efgartigimod) between these two classes of drugs (39). Frauke Stascheit et al. assessed serum calprotectin and serum neurofilament light chain levels in a total of 22 AChR antibody-positive gMG patients who were treatment-naive for either complement C5 inhibitors or FcRn inhibitors, both efgartigimod and ravulizumab were found to have comparable levels of MG-related biomarker dynamics in patients (40). Based on existing research, we speculate that these discrepancies may be associated with baseline characteristics, data source and reporting bias. Certainly, the risks and benefits of these two classes of drugs for MG warrant further evaluation.

### 4.4 SOCs associated with positive AE signals

In the SOC mapping analysis, we identified that nervous system disorders, general disorders and administration site conditions, musculoskeletal and connective tissue disorders were AEs common to both complement C5 inhibitors and FcRn inhibitors, which may be related to the pathophysiology of MG itself. Hehir MK et al. described in a review that patients with generalized MG exhibit varying degrees of fatigable weakness in ocular, bulbar, respiratory, axial and limb muscles, leading to clinical manifestations including ptosis, diplopia, bulbar dysfunction, chewing difficulty, dysphagia, bilateral facial paralysis, dyspnea, amyotrophic lateral sclerosis and limb weakness (41). Additionally, infections and infestations were observed in both drug classes. The prescribing information for complement C5 inhibitors specifically warns about meningococcal infections, while FcRn inhibitors are associated with respiratory tract infections, urinary tract infections, and herpes zoster. These AEs have also been reported in related studies (42–45). Beyond these shared characteristics, some SOCs deserve attention, including vascular disorders and respiratory, thoracic, and mediastinal disorders (complement C5 inhibitors), gastrointestinal disorders, metabolism and nutrition disorders, and psychiatric disorders (FcRn inhibitors). We further investigated the mechanisms underlying these SOC specificities for both drug classes.

### 4.5 Potential toxicological mechanisms of AEs

We found that several enriched signaling pathways in our toxicological study were potentially associated with novel AEs induced by complement C5 and FcRn inhibitors. A correlation exists between eculizumab’s newly discovered AE of embolic stroke and the complement and coagulation cascades. Michał Ząbczyk et al. found that embolic stroke is associated with proteins involved in the complement and coagulation cascades (46). The pathways for diabetic complications (47) and complement and coagulation cascades (48) may be involved in ravulizumab-induced hypoacusis. Changes in body weight in zilucoplan patients may be attributed to regulatory effects on NF-κB-enriched pathways (49). In addition, the newly identified AE of angina pectoris associated with efgartigimod may be regulated by the cytokine-cytokine receptor interaction pathway (50).

## 5 Limitations

Although the signal mining results based on the large sample data of FAERS can provide important references for clinical drug safety research, there are still some limitations in the study: 1. Data Source and Reporting Bias: Due to the inherent characteristics of the FAERS database, there are problems such as non-standard data reporting, underreporting, and missing clinical information of patients, which may lead to the underestimation or overestimation of AE signals. For example, symptoms such as fatigue or dyspnea, which may reflect underlying MG rather than adverse effects of the drug, should not be reported as AEs. Reports from this database were mainly from the United States, with a relatively low proportion of data from Asian populations, which may have contributed to ethnic differences in the research results. Moreover, among all the reports, the reports submitted by consumers accounted for a larger proportion than those from medical professionals. It could lead to a lack of professionalism in the reports, thereby affecting the quality of the data. 2. Limited Causal Inference: The signals obtained through the disproportionality method only indicate a statistical association between the drug and the adverse reaction, but cannot determine the causal relationship. These associations still need to be verified through strict clinical research and in-depth evaluation. 3. Subgroup Analysis Limitations: In subgroup analyses based on factors such as gender and reporters, there may be an unequal distribution of sample sizes, which could affect the generalizability and reliability of the results.

## 6 Conclusion

In conclusion, this study investigated the AEs of complement C5 inhibitors and FcRn inhibitors and identified some new potential AE signals by pharmacovigilance method, and preliminarily revealed the toxicological mechanism of these five agents’ AEs by network toxicology method, which provided a reference for the selection of drugs for treating MG. Furthermore, the indication-restricted analytical approach established herein offers a new framework for comparative safety assessment of therapeutics within the same disease context.

## Data Availability

The original contributions presented in the study are included in the article/**Supplementary Material**. Further inquiries can be directed to the corresponding authors.
