# Supplementary material for: Safety profile of complement C5 inhibitors and FcRn inhibitors in the treatment of myasthenia gravis: analysis of the FAERS database and disease-gene interaction network
Source: Front Immunol. 2025 Oct 8;16:1667249. doi: 10.3389/fimmu.2025.1667249 (PMC12540168; doi:10.3389/fimmu.2025.1667249)
Supplement: Supplementary file 1 [file DataSheet1.docx]

Supplementary Figure 1 Listing the top 10 PTs of complement C5 inhibitors and analyzing the association with gender.


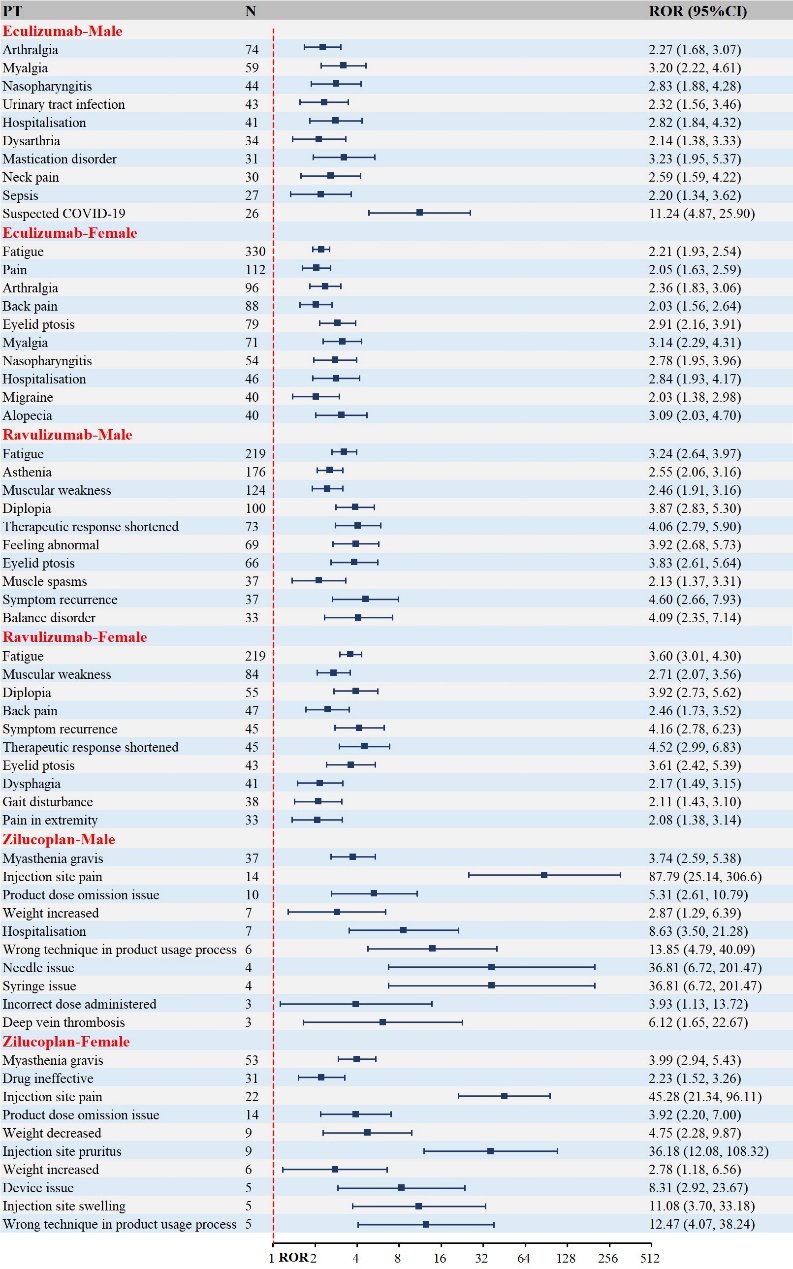


Abbreviation: PT: preferred terms; ROR: Reporting odds ratio.

Supplementary Figure 2 Listing the top 10 PTs of FcRn inhibitors and analyzing the association with gender.


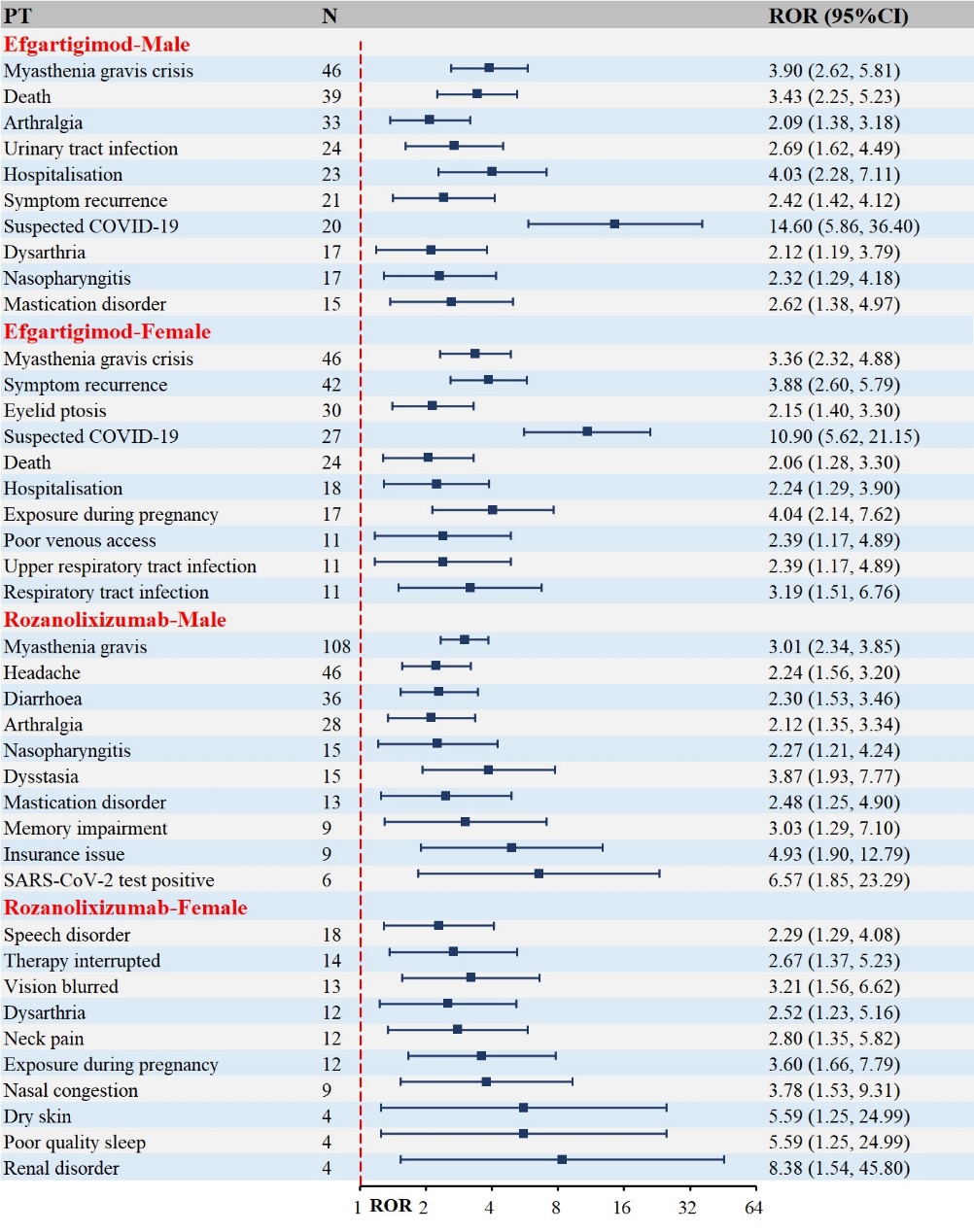


Abbreviation: PT: preferred terms; ROR: Reporting odds ratio.

Supplementary Figure 3 Listing the top 10 PTs of complement C5 inhibitors and analyzing the association with reporters.


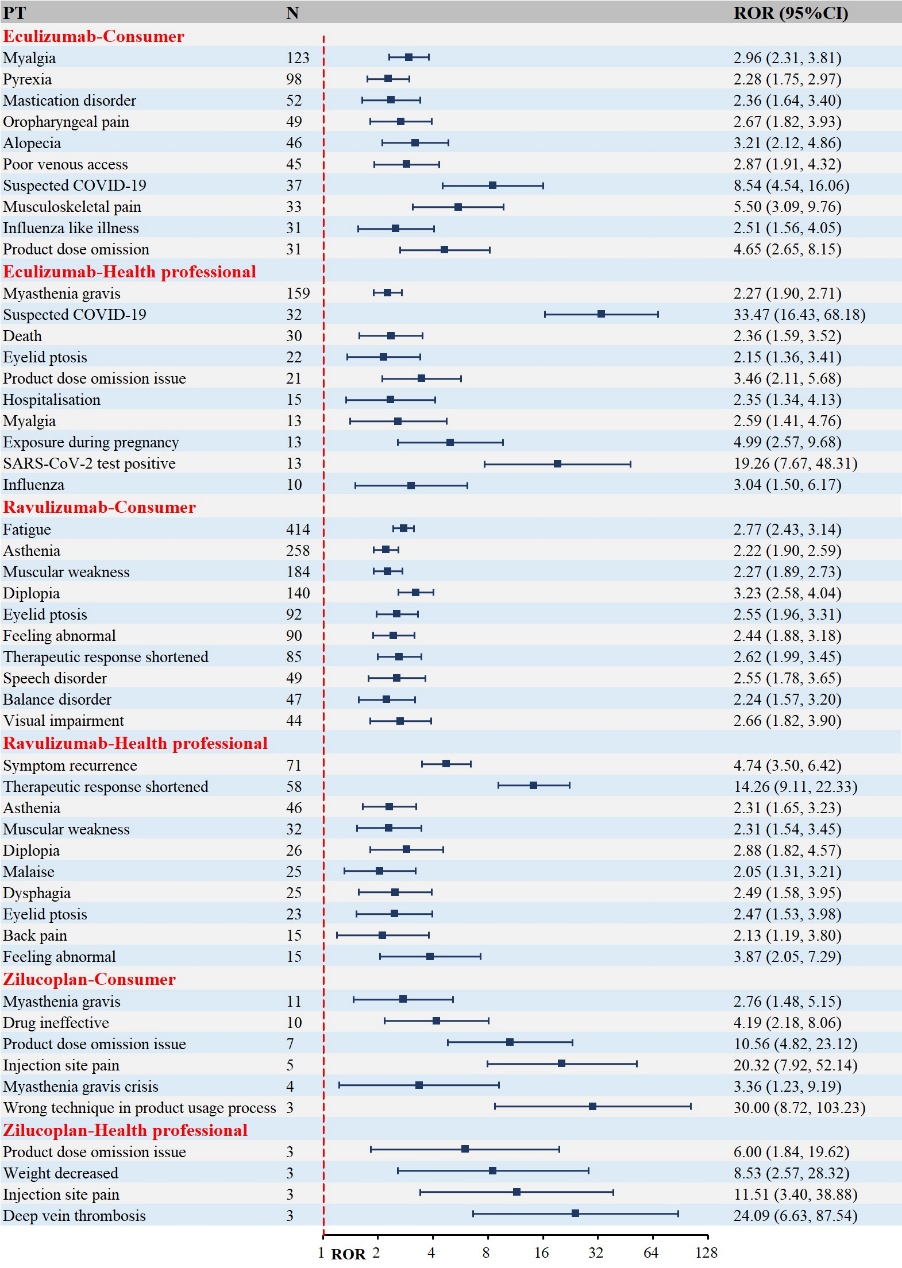


Abbreviation: PT: preferred terms; ROR: Reporting odds ratio.

Supplementary Figure 4 Listing the top 10 PTs of FcRn inhibitors and analyzing the association with reporters.


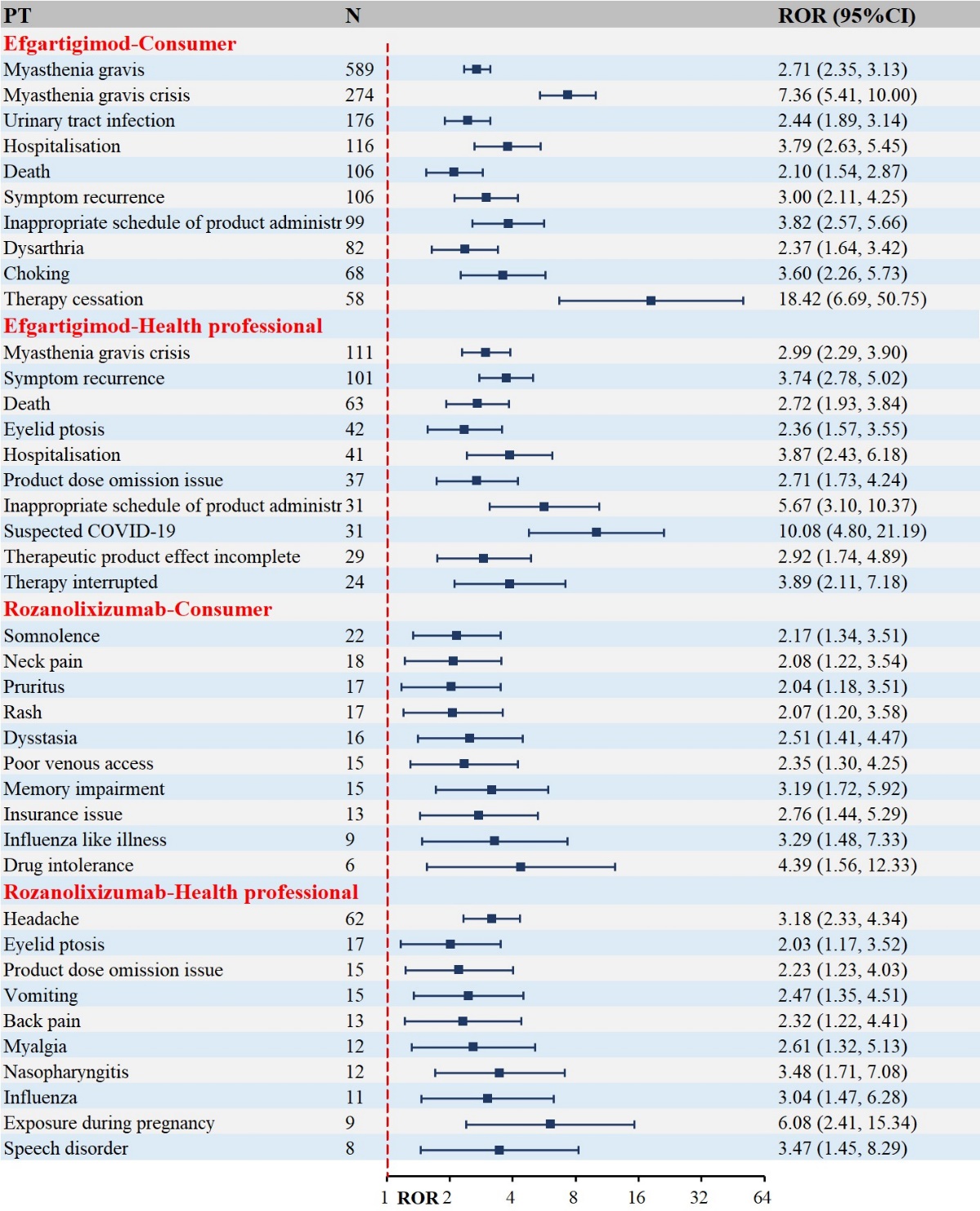


Abbreviation: PT: preferred terms; ROR: Reporting odds ratio.
