# Supplementary material for: Safety profile of complement C5 inhibitors and FcRn inhibitors in the treatment of myasthenia gravis: analysis of the FAERS database and disease-gene interaction network
Source: Front Immunol. 2025 Oct 8;16:1667249. doi: 10.3389/fimmu.2025.1667249 (PMC12540168; doi:10.3389/fimmu.2025.1667249)
Supplement: Supplementary file 2 [file DataSheet2.docx]

Supplementary Table 1 Two-by-two contingency table for disproportionality analyses.

| Types of Drugs | Target Adverse Event Reports of MG | Other Adverse Event Reports of MG | Total |
| --- | --- | --- | --- |
| Target drug | a | b | a+b |
| Other drugs | c | d | c+d |
| Total | a+c | b+d | a+b+c+d |

Abbreviation: MG: myasthenia gravis

Supplementary Table 2 Three major algorithms used for signal detection.

| Algorithms | Formula | Threshold |
| --- | --- | --- |
| ROR | *ROR*=(a/c)/(b/d)  *SE*(ln*ROR*)=$\sqrt{\text{(1/a+1/b+1/c+1/d)}}$  95*%CI*=*e*^ln(^*^ROR^*^)±1.96^*^SE^* | a ≥ 3, lower limit of 95% *CI* > 1 |
| MHRA | *PRR*=[a/(a+b)]/[c/(c+d)]  *χ^2^*=[(ad-bc)^2^(a+b+c+d)]/[(a+b)(c+d)(a+c)(b+d)] | a ≥ 3, *PRR* ≥ 2, *χ^2^* ≥ 4 |
| BCPNN | *IC*=log_2_[*p(x,y)*]/[*p(x)p(y)*]=log_2_[a(a+b+c+d)]/[(a+b)(a+c)]  *IC*-2*SD*=*E(IC)*-2$\sqrt{\text{V(IC)}}$ | *IC025* > 0 |

Abbreviation: *ROR*: Reporting odds ratio; *PRR*: Proportional reporting ratio; *CI*: Confidence interval; *IC*: Information component; *IC025*: The lower limit of the 95%*CI* of *IC*; *SE*: Standard error; *p(x, y)* : The probability of the combined occurrence of the target drug and the target adverse event; *p(x)* : The probability of using the target drug; *p(y)* : The probability of the target adverse event occurring; *SD*: Standard deviation; *E(IC)* : Expected value of the information component; *V(IC)* : Variance

Supplementary Table 3 PTs and corresponding SOCs with positive signals from all three methods for eculizumab.

| **PT** | **SOC** | **N** | **ROR** | **95%CI lower** | **95%CI upper** | **PRR** | **Chi2** | **IC** | **IC025** |
| --- | --- | --- | --- | --- | --- | --- | --- | --- | --- |
| Arthralgia | Musculoskeletal and connective tissue disorders | 185 | 2.18 | 1.83 | 2.60 | 2.16 | 77.45 | 0.83 | 0.59 |
| Myalgia | Musculoskeletal and connective tissue disorders | 140 | 3.09 | 2.48 | 3.84 | 3.07 | 113.91 | 1.14 | 0.84 |
| Suspected COVID-19 | Infections and infestations | 69 | 12.33 | 7.75 | 19.63 | 12.27 | 184.63 | 1.97 | 1.46 |
| Mastication disorder | Musculoskeletal and connective tissue disorders | 63 | 3.17 | 2.29 | 4.40 | 3.16 | 53.67 | 1.17 | 0.73 |
| Neck pain | Musculoskeletal and connective tissue disorders | 58 | 2.41 | 1.75 | 3.33 | 2.40 | 30.50 | 0.93 | 0.49 |
| Poor venous access | Vascular disorders | 51 | 2.66 | 1.88 | 3.78 | 2.66 | 32.51 | 1.02 | 0.55 |
| Oropharyngeal pain | Respiratory, thoracic and mediastinal disorders | 49 | 2.69 | 1.88 | 3.84 | 2.68 | 31.80 | 1.02 | 0.54 |
| Alopecia | Skin and subcutaneous tissue disorders | 49 | 2.80 | 1.95 | 4.01 | 2.79 | 34.08 | 1.06 | 0.58 |
| Rhinorrhoea | Respiratory, thoracic and mediastinal disorders | 44 | 2.30 | 1.59 | 3.31 | 2.29 | 20.86 | 0.88 | 0.39 |
| Musculoskeletal pain | Musculoskeletal and connective tissue disorders | 36 | 6.42 | 3.83 | 10.76 | 6.40 | 65.72 | 1.66 | 1.02 |
| Memory impairment | Nervous system disorders | 35 | 2.11 | 1.41 | 3.16 | 2.11 | 13.61 | 0.80 | 0.26 |
| Influenza like illness | General disorders and administration site conditions | 33 | 2.14 | 1.41 | 3.25 | 2.14 | 13.29 | 0.81 | 0.26 |
| Product dose omission | Injury, poisoning and procedural complications | 32 | 4.15 | 2.55 | 6.75 | 4.14 | 38.72 | 1.38 | 0.74 |
| SARS-CoV-2 test positive | Investigations | 31 | 4.42 | 2.67 | 7.30 | 4.41 | 40.26 | 1.42 | 0.77 |
| Exposure during pregnancy | Injury, poisoning and procedural complications | 30 | 3.47 | 2.14 | 5.61 | 3.46 | 29.03 | 1.24 | 0.60 |
| Adverse event | General disorders and administration site conditions | 29 | 4.77 | 2.81 | 8.10 | 4.76 | 40.77 | 1.47 | 0.79 |
| Bone pain | Musculoskeletal and connective tissue disorders | 28 | 4.13 | 2.46 | 6.94 | 4.12 | 33.71 | 1.37 | 0.70 |
| Plasmapheresis | Surgical and medical procedures | 27 | 5.25 | 2.99 | 9.22 | 5.24 | 41.61 | 1.54 | 0.82 |
| White blood cell count decreased | Investigations | 24 | 2.63 | 1.58 | 4.38 | 2.63 | 14.99 | 1.01 | 0.33 |
| Aphasia | Nervous system disorders | 19 | 2.03 | 1.18 | 3.51 | 2.03 | 6.72 | 0.76 | 0.05 |
| Fluid retention | Metabolism and nutrition disorders | 17 | 2.34 | 1.30 | 4.23 | 2.34 | 8.44 | 0.90 | 0.13 |
| Eating disorder | Psychiatric disorders | 15 | 2.47 | 1.31 | 4.65 | 2.46 | 8.27 | 0.95 | 0.12 |
| Blister | Skin and subcutaneous tissue disorders | 15 | 3.05 | 1.57 | 5.92 | 3.05 | 12.06 | 1.14 | 0.28 |
| Poor quality sleep | Psychiatric disorders | 13 | 2.52 | 1.27 | 5.01 | 2.52 | 7.51 | 0.97 | 0.09 |
| Inappropriate schedule of drug administration | Injury, poisoning and procedural complications | 13 | 27.78 | 6.27 | 123.10 | 27.75 | 44.71 | 2.19 | 1.06 |
| Central venous catheterisation | Surgical and medical procedures | 11 | 2.47 | 1.18 | 5.20 | 2.47 | 6.11 | 0.95 | 0.01 |
| Osteomyelitis | Infections and infestations | 11 | 3.62 | 1.62 | 8.07 | 3.61 | 11.26 | 1.27 | 0.27 |
| Ocular discomfort | Eye disorders | 11 | 3.92 | 1.73 | 8.88 | 3.91 | 12.45 | 1.33 | 0.32 |
| Pregnancy | Pregnancy, puerperium and perinatal conditions | 11 | 5.87 | 2.36 | 14.61 | 5.87 | 18.72 | 1.61 | 0.54 |
| Upper limb fracture | Injury, poisoning and procedural complications | 10 | 2.85 | 1.28 | 6.34 | 2.85 | 7.19 | 1.08 | 0.07 |
| Product preparation issue | Injury, poisoning and procedural complications | 8 | 4.27 | 1.60 | 11.38 | 4.27 | 10.02 | 1.40 | 0.22 |
| Drug effect decreased | General disorders and administration site conditions | 8 | 4.88 | 1.77 | 13.46 | 4.88 | 11.52 | 1.49 | 0.30 |
| Product preparation error | Injury, poisoning and procedural complications | 8 | 11.39 | 3.02 | 42.94 | 11.39 | 20.67 | 1.94 | 0.65 |
| General physical condition abnormal | Investigations | 7 | 3.32 | 1.24 | 8.92 | 3.32 | 6.39 | 1.21 | 0.01 |
| Escherichia urinary tract infection | Infections and infestations | 7 | 4.27 | 1.50 | 12.18 | 4.27 | 8.76 | 1.40 | 0.16 |
| Pyelonephritis | Infections and infestations | 7 | 4.98 | 1.67 | 14.83 | 4.98 | 10.28 | 1.51 | 0.24 |
| Intervertebral disc degeneration | Musculoskeletal and connective tissue disorders | 6 | 4.27 | 1.38 | 13.24 | 4.27 | 7.51 | 1.40 | 0.09 |
| Catheter site pain | General disorders and administration site conditions | 6 | 5.13 | 1.56 | 16.80 | 5.12 | 9.05 | 1.52 | 0.18 |
| Product temperature excursion issue | Injury, poisoning and procedural complications | 6 | 5.13 | 1.56 | 16.80 | 5.12 | 9.05 | 1.52 | 0.18 |
| Posture abnormal | Musculoskeletal and connective tissue disorders | 6 | 6.41 | 1.81 | 22.71 | 6.40 | 10.95 | 1.66 | 0.29 |
| General symptom | General disorders and administration site conditions | 6 | 25.63 | 3.09 | 212.88 | 25.62 | 20.28 | 2.18 | 0.69 |
| Meningitis cryptococcal | Infections and infestations | 5 | 7.12 | 1.70 | 29.79 | 7.12 | 9.86 | 1.72 | 0.25 |
| Antibody test abnormal | Investigations | 5 | 10.68 | 2.07 | 55.04 | 10.67 | 12.53 | 1.91 | 0.40 |
| Gastric cancer | Neoplasms benign, malignant and unspecified | 5 | 21.35 | 2.49 | 182.80 | 21.35 | 16.16 | 2.14 | 0.57 |
| Extraocular muscle paresis | Eye disorders | 4 | 5.69 | 1.27 | 25.44 | 5.69 | 6.63 | 1.59 | 0.04 |
| Full blood count decreased | Investigations | 4 | 5.69 | 1.27 | 25.44 | 5.69 | 6.63 | 1.59 | 0.04 |
| Iron deficiency | Metabolism and nutrition disorders | 4 | 5.69 | 1.27 | 25.44 | 5.69 | 6.63 | 1.59 | 0.04 |
| Meningococcal sepsis | Infections and infestations | 4 | 8.54 | 1.56 | 46.64 | 8.54 | 8.88 | 1.81 | 0.21 |
| Middle ear effusion | Ear and labyrinth disorders | 4 | 8.54 | 1.56 | 46.64 | 8.54 | 8.88 | 1.81 | 0.21 |
| Escherichia sepsis | Infections and infestations | 4 | 17.08 | 1.91 | 152.85 | 17.08 | 12.11 | 2.08 | 0.42 |
| Multimorbidity | General disorders and administration site conditions | 4 | 17.08 | 1.91 | 152.85 | 17.08 | 12.11 | 2.08 | 0.42 |
| Axillary mass | Musculoskeletal and connective tissue disorders | 3 | 12.81 | 1.33 | 123.17 | 12.81 | 8.17 | 1.98 | 0.21 |
| Blood creatinine abnormal | Investigations | 3 | 12.81 | 1.33 | 123.17 | 12.81 | 8.17 | 1.98 | 0.21 |
| Embolic stroke | Nervous system disorders | 3 | 12.81 | 1.33 | 123.17 | 12.81 | 8.17 | 1.98 | 0.21 |
| Eye symptom | Eye disorders | 3 | 12.81 | 1.33 | 123.17 | 12.81 | 8.17 | 1.98 | 0.21 |
| Red cell distribution width increased | Investigations | 3 | 12.81 | 1.33 | 123.17 | 12.81 | 8.17 | 1.98 | 0.21 |
| Vascular graft complication | Injury, poisoning and procedural complications | 3 | 12.81 | 1.33 | 123.17 | 12.81 | 8.17 | 1.98 | 0.21 |
| Aeromonas infection | Infections and infestations | 3 |  |  |  | 29.88 | 12.21 | 2.21 | 0.46 |
| Asymptomatic COVID-19 | Infections and infestations | 3 |  |  |  | 29.88 | 12.21 | 2.21 | 0.46 |
| Breast cancer recurrent | Neoplasms benign, malignant and unspecified | 3 |  |  |  | 29.88 | 12.21 | 2.21 | 0.46 |
| Eating disorder symptom | Metabolism and nutrition disorders | 3 |  |  |  | 29.88 | 12.21 | 2.21 | 0.46 |
| Lumbosacral radiculopathy | Nervous system disorders | 3 |  |  |  | 29.88 | 12.21 | 2.21 | 0.46 |
| Neuromyelitis optica spectrum disorder | Nervous system disorders | 3 |  |  |  | 29.88 | 12.21 | 2.21 | 0.46 |
| Pustule | Infections and infestations | 3 |  |  |  | 29.88 | 12.21 | 2.21 | 0.46 |
| Retinal haemorrhage | Eye disorders | 3 |  |  |  | 29.88 | 12.21 | 2.21 | 0.46 |
| Streptococcal urinary tract infection | Infections and infestations | 3 |  |  |  | 29.88 | 12.21 | 2.21 | 0.46 |

Abbreviation: PT: preferred terms; SOC: system organ categories; ROR: Reporting odds ratio; PRR: Proportional reporting ratio; IC: Information component; IC025: The lower limit of the 95%CI of IC.

Supplementary Table 4 PTs and corresponding SOCs with positive signals from all three methods for ravulizumab.

| **PT** | **SOC** | **N** | **ROR** | **95%CI lower** | **95%CI upper** | **PRR** | **Chi2** | **IC** | **IC025** |
| --- | --- | --- | --- | --- | --- | --- | --- | --- | --- |
| Fatigue | General disorders and administration site conditions | 584 | 2.73 | 2.45 | 3.04 | 2.62 | 360.27 | 0.98 | 0.84 |
| Asthenia | General disorders and administration site conditions | 382 | 2.29 | 2.02 | 2.61 | 2.24 | 169.52 | 0.84 | 0.67 |
| Muscular weakness | Musculoskeletal and connective tissue disorders | 260 | 2.29 | 1.96 | 2.68 | 2.26 | 116.56 | 0.84 | 0.64 |
| Diplopia | Eye disorders | 193 | 3.00 | 2.48 | 3.62 | 2.96 | 142.76 | 1.08 | 0.83 |
| Therapeutic response shortened | General disorders and administration site conditions | 186 | 4.49 | 3.63 | 5.54 | 4.42 | 230.58 | 1.37 | 1.10 |
| Eyelid ptosis | Eye disorders | 149 | 2.69 | 2.18 | 3.32 | 2.67 | 92.34 | 0.99 | 0.71 |
| Feeling abnormal | General disorders and administration site conditions | 134 | 2.77 | 2.21 | 3.46 | 2.74 | 87.18 | 1.01 | 0.72 |
| Symptom recurrence | General disorders and administration site conditions | 125 | 2.23 | 1.79 | 2.79 | 2.22 | 53.31 | 0.83 | 0.54 |
| Back pain | Musculoskeletal and connective tissue disorders | 124 | 2.02 | 1.62 | 2.51 | 2.00 | 41.34 | 0.73 | 0.45 |
| Gait disturbance | General disorders and administration site conditions | 118 | 2.20 | 1.75 | 2.76 | 2.18 | 48.56 | 0.81 | 0.51 |
| Visual impairment | Eye disorders | 64 | 2.75 | 1.99 | 3.79 | 2.74 | 41.32 | 1.01 | 0.59 |
| Balance disorder | Nervous system disorders | 63 | 2.36 | 1.73 | 3.23 | 2.35 | 30.52 | 0.88 | 0.47 |
| Illness | General disorders and administration site conditions | 62 | 2.14 | 1.57 | 2.92 | 2.13 | 24.00 | 0.79 | 0.38 |
| Speech disorder | Nervous system disorders | 60 | 2.36 | 1.71 | 3.26 | 2.36 | 29.12 | 0.88 | 0.46 |
| Somnolence | Nervous system disorders | 56 | 2.57 | 1.83 | 3.61 | 2.56 | 32.17 | 0.96 | 0.51 |
| General physical health deterioration | General disorders and administration site conditions | 51 | 3.40 | 2.33 | 4.95 | 3.38 | 45.65 | 1.18 | 0.69 |
| Neck pain | Musculoskeletal and connective tissue disorders | 45 | 2.99 | 2.03 | 4.42 | 2.98 | 33.51 | 1.08 | 0.58 |
| Mastication disorder | Musculoskeletal and connective tissue disorders | 44 | 2.88 | 1.95 | 4.25 | 2.87 | 30.75 | 1.05 | 0.54 |
| Stress | Psychiatric disorders | 44 | 2.29 | 1.58 | 3.33 | 2.29 | 20.04 | 0.85 | 0.37 |
| Musculoskeletal stiffness | Musculoskeletal and connective tissue disorders | 39 | 2.69 | 1.78 | 4.05 | 2.68 | 24.24 | 0.99 | 0.46 |
| Eye disorder | Eye disorders | 36 | 3.65 | 2.32 | 5.77 | 3.64 | 35.53 | 1.24 | 0.65 |
| Drug effect less than expected | General disorders and administration site conditions | 33 | 2.40 | 1.55 | 3.71 | 2.40 | 16.56 | 0.90 | 0.33 |
| Musculoskeletal discomfort | Musculoskeletal and connective tissue disorders | 30 | 3.86 | 2.32 | 6.40 | 3.85 | 31.64 | 1.28 | 0.64 |
| Limb discomfort | Musculoskeletal and connective tissue disorders | 29 | 2.24 | 1.41 | 3.53 | 2.23 | 12.49 | 0.83 | 0.24 |
| Asthenopia | Eye disorders | 28 | 5.68 | 3.17 | 10.18 | 5.67 | 43.57 | 1.53 | 0.83 |
| Memory impairment | Nervous system disorders | 27 | 2.21 | 1.38 | 3.56 | 2.21 | 11.38 | 0.82 | 0.21 |
| Arthritis | Musculoskeletal and connective tissue disorders | 24 | 2.01 | 1.23 | 3.29 | 2.01 | 7.98 | 0.73 | 0.10 |
| Muscle fatigue | Musculoskeletal and connective tissue disorders | 21 | 3.52 | 1.95 | 6.36 | 3.51 | 19.74 | 1.21 | 0.46 |
| Photophobia | Eye disorders | 20 | 5.14 | 2.63 | 10.04 | 5.13 | 28.51 | 1.47 | 0.66 |
| Brain fog | Nervous system disorders | 17 | 2.11 | 1.17 | 3.82 | 2.11 | 6.41 | 0.78 | 0.03 |
| Temperature intolerance | General disorders and administration site conditions | 16 | 4.74 | 2.28 | 9.86 | 4.73 | 21.14 | 1.42 | 0.54 |
| Muscle disorder | Musculoskeletal and connective tissue disorders | 15 | 4.81 | 2.25 | 10.29 | 4.81 | 20.12 | 1.43 | 0.52 |
| Hypoacusis | Ear and labyrinth disorders | 13 | 3.13 | 1.50 | 6.51 | 3.13 | 10.37 | 1.12 | 0.21 |
| Nerve compression | Nervous system disorders | 11 | 4.24 | 1.80 | 9.98 | 4.23 | 12.93 | 1.34 | 0.32 |
| Sluggishness | General disorders and administration site conditions | 10 | 2.96 | 1.30 | 6.76 | 2.96 | 7.33 | 1.08 | 0.07 |
| Dropped head syndrome | Nervous system disorders | 10 | 4.81 | 1.90 | 12.20 | 4.81 | 13.41 | 1.43 | 0.35 |
| Hypokinesia | Nervous system disorders | 10 | 4.81 | 1.90 | 12.20 | 4.81 | 13.41 | 1.43 | 0.35 |
| Restless legs syndrome | Nervous system disorders | 9 | 2.89 | 1.22 | 6.85 | 2.89 | 6.34 | 1.06 | 0.00 |
| Ophthalmoplegia | Eye disorders | 8 | 6.16 | 2.01 | 18.83 | 6.16 | 13.29 | 1.58 | 0.36 |
| Facial paresis | Nervous system disorders | 8 | 3.42 | 1.32 | 8.87 | 3.42 | 7.25 | 1.19 | 0.06 |
| General physical condition abnormal | Investigations | 7 | 4.49 | 1.51 | 13.37 | 4.49 | 8.76 | 1.38 | 0.15 |
| Jaw disorder | Musculoskeletal and connective tissue disorders | 7 | 6.74 | 1.97 | 23.02 | 6.73 | 12.43 | 1.63 | 0.34 |
| Thyroid disorder | Endocrine disorders | 6 | 4.62 | 1.41 | 15.14 | 4.62 | 7.73 | 1.40 | 0.09 |
| Sleep deficit | Nervous system disorders | 6 | 5.77 | 1.63 | 20.46 | 5.77 | 9.47 | 1.54 | 0.20 |
| Frustration tolerance decreased | Psychiatric disorders | 6 | 5.77 | 1.63 | 20.46 | 5.77 | 9.47 | 1.54 | 0.20 |
| Cyst | General disorders and administration site conditions | 5 | 6.41 | 1.53 | 26.84 | 6.41 | 8.57 | 1.60 | 0.15 |
| Meningococcal infection | Infections and infestations | 5 | 9.62 | 1.87 | 49.60 | 9.62 | 11.03 | 1.79 | 0.30 |
| Neisseria infection | Infections and infestations | 5 |  |  |  | 42.31 | 18.49 | 2.15 | 0.63 |
| Bone disorder | Musculoskeletal and connective tissue disorders | 5 | 4.81 | 1.29 | 17.92 | 4.81 | 6.70 | 1.43 | 0.02 |
| Thymoma malignant | Neoplasms benign, malignant and unspecified | 5 |  |  |  | 42.31 | 18.49 | 2.15 | 0.63 |
| Paranasal sinus hypersecretion | Respiratory, thoracic and mediastinal disorders | 4 | 15.39 | 1.72 | 137.74 | 15.39 | 10.76 | 1.96 | 0.32 |
| Altered visual depth perception | Eye disorders | 3 |  |  |  | 26.92 | 10.92 | 2.08 | 0.36 |
| Herpes simplex | Infections and infestations | 3 | 11.54 | 1.20 | 110.99 | 11.54 | 7.22 | 1.86 | 0.11 |
| International normalised ratio increased | Investigations | 3 | 11.54 | 1.20 | 110.99 | 11.54 | 7.22 | 1.86 | 0.11 |
| Mean platelet volume decreased | Investigations | 3 |  |  |  | 26.92 | 10.92 | 2.08 | 0.36 |
| Intervertebral disc disorder | Musculoskeletal and connective tissue disorders | 3 | 11.54 | 1.20 | 110.99 | 11.54 | 7.22 | 1.86 | 0.11 |
| Psoriatic arthropathy | Musculoskeletal and connective tissue disorders | 3 | 11.54 | 1.20 | 110.99 | 11.54 | 7.22 | 1.86 | 0.11 |
| Trigger finger | Musculoskeletal and connective tissue disorders | 3 | 11.54 | 1.20 | 110.99 | 11.54 | 7.22 | 1.86 | 0.11 |
| Nocturnal dyspnoea | Respiratory, thoracic and mediastinal disorders | 3 | 11.54 | 1.20 | 110.99 | 11.54 | 7.22 | 1.86 | 0.11 |
| Upper respiratory tract congestion | Respiratory, thoracic and mediastinal disorders | 3 | 11.54 | 1.20 | 110.99 | 11.54 | 7.22 | 1.86 | 0.11 |
| Peripheral vascular disorder | Vascular disorders | 3 | 11.54 | 1.20 | 110.99 | 11.54 | 7.22 | 1.86 | 0.11 |

Abbreviation: PT: preferred terms; SOC: system organ categories; ROR: Reporting odds ratio; PRR: Proportional reporting ratio; IC: Information component; IC025: The lower limit of the 95%CI of IC.

Supplementary Table 5 PTs and corresponding SOCs with positive signals from all three methods for zilucoplan.

| **PT** | **SOC** | **N** | **ROR** | **95%CI lower** | **95%CI upper** | **PRR** | **Chi2** | **IC** | **IC025** |
| --- | --- | --- | --- | --- | --- | --- | --- | --- | --- |
| Myasthenia gravis | Nervous system disorders | 91 | 2.58 | 2.06 | 3.22 | 2.44 | 74.57 | 1.23 | 0.85 |
| Drug ineffective | General disorders and administration site conditions | 58 | 2.55 | 1.94 | 3.35 | 2.46 | 47.86 | 1.24 | 0.77 |
| Injection site pain | General disorders and administration site conditions | 36 | 30.27 | 19.21 | 47.69 | 29.24 | 517.86 | 3.99 | 2.86 |
| Product dose omission issue | Injury, poisoning and procedural complications | 24 | 4.65 | 3.02 | 7.15 | 4.56 | 58.82 | 2.04 | 1.20 |
| Weight increased | Investigations | 13 | 3.00 | 1.69 | 5.31 | 2.97 | 15.68 | 1.49 | 0.51 |
| Weight decreased | Investigations | 11 | 2.52 | 1.36 | 4.66 | 2.50 | 9.23 | 1.26 | 0.26 |
| Hospitalisation | Surgical and medical procedures | 11 | 2.81 | 1.51 | 5.22 | 2.79 | 11.70 | 1.41 | 0.37 |
| Wrong technique in product usage process | Injury, poisoning and procedural complications | 11 | 16.41 | 7.94 | 33.93 | 16.24 | 105.00 | 3.48 | 1.98 |
| Injection site pruritus | General disorders and administration site conditions | 10 | 32.80 | 13.62 | 78.97 | 32.49 | 152.67 | 4.07 | 2.41 |
| Syringe issue | Product issues | 8 | 65.47 | 19.68 | 217.79 | 64.97 | 168.02 | 4.48 | 2.68 |
| Device issue | Product issues | 7 | 14.30 | 5.87 | 34.84 | 14.21 | 59.85 | 3.35 | 1.69 |
| Injection site bruising | General disorders and administration site conditions | 7 | 22.89 | 8.70 | 60.26 | 22.74 | 85.62 | 3.79 | 2.05 |
| Incorrect dose administered | Injury, poisoning and procedural complications | 6 | 3.92 | 1.68 | 9.15 | 3.90 | 11.56 | 1.84 | 0.43 |
| Therapeutic response decreased | General disorders and administration site conditions | 6 | 4.66 | 1.98 | 10.99 | 4.64 | 15.02 | 2.07 | 0.61 |
| Intentional dose omission | Injury, poisoning and procedural complications | 6 | 8.52 | 3.46 | 20.97 | 8.47 | 31.40 | 2.79 | 1.18 |
| Needle issue | Product issues | 6 | 49.01 | 13.81 | 173.94 | 48.73 | 112.23 | 4.33 | 2.46 |
| Injection site swelling | General disorders and administration site conditions | 5 | 7.09 | 2.69 | 18.69 | 7.06 | 21.39 | 2.58 | 0.93 |
| Injection site rash | General disorders and administration site conditions | 5 | 10.88 | 3.95 | 29.98 | 10.83 | 33.47 | 3.07 | 1.32 |
| Injection site erythema | General disorders and administration site conditions | 4 | 3.62 | 1.29 | 10.19 | 3.61 | 6.80 | 1.74 | 0.16 |
| Respiratory disorder | Respiratory, thoracic and mediastinal disorders | 4 | 3.72 | 1.32 | 10.50 | 3.71 | 7.12 | 1.78 | 0.19 |
| Adverse drug reaction | General disorders and administration site conditions | 4 | 3.83 | 1.36 | 10.82 | 3.82 | 7.47 | 1.82 | 0.22 |
| Deep vein thrombosis | Vascular disorders | 4 | 7.67 | 2.58 | 22.83 | 7.64 | 18.71 | 2.67 | 0.90 |
| Injection site mass | General disorders and administration site conditions | 4 | 130.45 | 14.57 | 1168.18 | 129.94 | 102.37 | 4.74 | 2.72 |
| Viral infection | Infections and infestations | 3 | 3.76 | 1.14 | 12.43 | 3.75 | 5.43 | 1.79 | 0.06 |
| Back disorder | Musculoskeletal and connective tissue disorders | 3 | 5.43 | 1.60 | 18.45 | 5.41 | 9.26 | 2.26 | 0.44 |
| Bedridden | Social circumstances | 3 | 6.11 | 1.78 | 20.99 | 6.09 | 10.76 | 2.40 | 0.56 |
| Gastroenteritis viral | Infections and infestations | 3 | 6.11 | 1.78 | 20.99 | 6.09 | 10.76 | 2.40 | 0.56 |
| Plasmapheresis | Surgical and medical procedures | 3 | 8.14 | 2.29 | 28.90 | 8.12 | 14.99 | 2.74 | 0.84 |
| Product storage error | Injury, poisoning and procedural complications | 3 | 10.86 | 2.94 | 40.17 | 10.83 | 20.08 | 3.07 | 1.12 |
| Therapeutic response unexpected | General disorders and administration site conditions | 3 | 12.22 | 3.24 | 46.11 | 12.18 | 22.40 | 3.19 | 1.23 |
| Knee operation | Surgical and medical procedures | 3 | 16.29 | 4.07 | 65.22 | 16.24 | 28.62 | 3.48 | 1.48 |
| Injection site irritation | General disorders and administration site conditions | 3 | 48.87 | 8.16 | 292.79 | 48.73 | 56.11 | 4.33 | 2.25 |
| Injection site induration | General disorders and administration site conditions | 3 | 97.74 | 10.16 | 940.47 | 97.46 | 71.61 | 4.65 | 2.56 |

Abbreviation: PT: preferred terms; SOC: system organ categories; ROR: Reporting odds ratio; PRR: Proportional reporting ratio; IC: Information component; IC025: The lower limit of the 95%CI of IC.

Supplementary Table 6 PTs and corresponding SOCs with positive signals from all three methods for zilucoplan.

| **PT** | **SOC** | **N** | **ROR** | **95%CI lower** | **95%CI upper** | **PRR** | **Chi2** | **IC** | **IC025** |
| --- | --- | --- | --- | --- | --- | --- | --- | --- | --- |
| Myasthenia gravis | Nervous system disorders | 694 | 2.16 | 1.96 | 2.39 | 2.10 | 238.30 | 0.71 | 0.59 |
| Myasthenia gravis crisis | Nervous system disorders | 346 | 4.78 | 4.02 | 5.67 | 4.67 | 384.93 | 1.26 | 1.07 |
| Urinary tract infection | Infections and infestations | 188 | 2.61 | 2.14 | 3.18 | 2.59 | 97.17 | 0.88 | 0.64 |
| Symptom recurrence | General disorders and administration site conditions | 173 | 2.87 | 2.32 | 3.54 | 2.84 | 104.72 | 0.95 | 0.69 |
| Hospitalisation | Surgical and medical procedures | 132 | 3.99 | 3.06 | 5.19 | 3.96 | 123.28 | 1.17 | 0.86 |
| Inappropriate schedule of product administration | Injury, poisoning and procedural complications | 126 | 5.15 | 3.84 | 6.89 | 5.11 | 150.48 | 1.31 | 0.99 |
| Therapy interrupted | Surgical and medical procedures | 84 | 3.79 | 2.74 | 5.26 | 3.78 | 74.33 | 1.14 | 0.76 |
| Dysarthria | Nervous system disorders | 75 | 2.23 | 1.65 | 3.02 | 2.22 | 28.59 | 0.76 | 0.39 |
| Choking | Respiratory, thoracic and mediastinal disorders | 73 | 4.59 | 3.17 | 6.64 | 4.57 | 78.74 | 1.25 | 0.83 |
| Therapy cessation | Surgical and medical procedures | 68 | 17.87 | 9.45 | 33.80 | 17.78 | 150.20 | 1.74 | 1.25 |
| Procedural headache | Injury, poisoning and procedural complications | 86 |  |  |  | 497.58 | 246.81 | 1.95 | 1.49 |
| Upper respiratory tract infection | Infections and infestations | 54 | 2.40 | 1.67 | 3.44 | 2.39 | 23.89 | 0.82 | 0.38 |
| Nephrolithiasis | Renal and urinary disorders | 52 | 4.29 | 2.79 | 6.58 | 4.27 | 52.55 | 1.21 | 0.73 |
| Therapeutic product effect decreased | General disorders and administration site conditions | 47 | 2.38 | 1.62 | 3.50 | 2.37 | 20.48 | 0.81 | 0.34 |
| Loss of personal independence in daily activities | Social circumstances | 44 | 2.95 | 1.94 | 4.49 | 2.94 | 27.95 | 0.97 | 0.47 |
| Cataract | Eye disorders | 34 | 2.13 | 1.37 | 3.32 | 2.13 | 11.68 | 0.72 | 0.18 |
| Dehydration | Metabolism and nutrition disorders | 34 | 2.39 | 1.52 | 3.77 | 2.39 | 14.98 | 0.81 | 0.27 |
| Dyspnoea at rest | Respiratory, thoracic and mediastinal disorders | 27 | 25.94 | 7.87 | 85.52 | 25.89 | 64.64 | 1.80 | 1.04 |
| Respiratory tract infection | Infections and infestations | 24 | 2.09 | 1.24 | 3.54 | 2.09 | 7.93 | 0.71 | 0.08 |
| Thymectomy | Surgical and medical procedures | 24 | 13.83 | 5.28 | 36.26 | 13.81 | 49.18 | 1.68 | 0.90 |
| Mechanical ventilation | Surgical and medical procedures | 22 | 12.68 | 4.80 | 33.48 | 12.66 | 43.76 | 1.66 | 0.85 |
| Injection site erythema | General disorders and administration site conditions | 21 | 2.52 | 1.40 | 4.53 | 2.52 | 10.25 | 0.86 | 0.16 |
| Respiratory syncytial virus infection | Infections and infestations | 21 | 3.56 | 1.88 | 6.75 | 3.55 | 17.25 | 1.10 | 0.37 |
| Respiratory disorder | Respiratory, thoracic and mediastinal disorders | 19 | 2.10 | 1.16 | 3.80 | 2.10 | 6.35 | 0.71 | 0.01 |
| Ill-defined disorder | General disorders and administration site conditions | 19 | 5.47 | 2.54 | 11.77 | 5.47 | 23.92 | 1.35 | 0.54 |
| Lacrimation increased | Eye disorders | 18 | 2.25 | 1.22 | 4.18 | 2.25 | 7.03 | 0.77 | 0.04 |
| Surgery | Surgical and medical procedures | 18 | 3.46 | 1.74 | 6.86 | 3.45 | 14.26 | 1.08 | 0.30 |
| Blepharospasm | Eye disorders | 16 | 2.56 | 1.30 | 5.02 | 2.56 | 8.04 | 0.87 | 0.08 |
| Cardiac failure congestive | Cardiac disorders | 15 | 2.54 | 1.27 | 5.09 | 2.54 | 7.43 | 0.86 | 0.05 |
| Eye pain | Eye disorders | 15 | 3.60 | 1.68 | 7.69 | 3.60 | 12.50 | 1.11 | 0.26 |
| Therapeutic product ineffective | General disorders and administration site conditions | 15 | 21.60 | 4.94 | 94.46 | 21.57 | 34.63 | 1.77 | 0.80 |
| Spinal operation | Surgical and medical procedures | 14 | 3.66 | 1.66 | 8.07 | 3.66 | 11.92 | 1.12 | 0.24 |
| Bulbar palsy | Nervous system disorders | 13 | 2.67 | 1.26 | 5.69 | 2.67 | 7.05 | 0.90 | 0.03 |
| Prostate cancer | Neoplasms benign, malignant and unspecified | 13 | 3.74 | 1.64 | 8.54 | 3.74 | 11.35 | 1.13 | 0.22 |
| Therapy change | Surgical and medical procedures | 13 | 4.16 | 1.78 | 9.73 | 4.16 | 12.75 | 1.20 | 0.27 |
| Injection site reaction | General disorders and administration site conditions | 13 | 4.68 | 1.94 | 11.29 | 4.67 | 14.31 | 1.26 | 0.33 |
| Endotracheal intubation | Surgical and medical procedures | 13 | 6.24 | 2.37 | 16.41 | 6.23 | 18.04 | 1.41 | 0.45 |
| Cataract operation | Surgical and medical procedures | 13 | 9.36 | 3.05 | 28.70 | 9.35 | 22.81 | 1.57 | 0.57 |
| Infusion site extravasation | General disorders and administration site conditions | 12 | 4.32 | 1.77 | 10.57 | 4.32 | 12.23 | 1.22 | 0.26 |
| Dysuria | Renal and urinary disorders | 11 | 3.17 | 1.34 | 7.46 | 3.16 | 7.76 | 1.02 | 0.06 |
| Musculoskeletal chest pain | Musculoskeletal and connective tissue disorders | 11 | 3.17 | 1.34 | 7.46 | 3.16 | 7.76 | 1.02 | 0.06 |
| Product storage error | Injury, poisoning and procedural complications | 11 | 4.52 | 1.75 | 11.67 | 4.52 | 11.73 | 1.24 | 0.24 |
| Knee arthroplasty | Surgical and medical procedures | 10 | 3.60 | 1.42 | 9.12 | 3.60 | 8.33 | 1.11 | 0.09 |
| Salivary hypersecretion | Gastrointestinal disorders | 10 | 3.60 | 1.42 | 9.12 | 3.60 | 8.33 | 1.11 | 0.09 |
| Physical deconditioning | General disorders and administration site conditions | 10 | 4.11 | 1.57 | 10.80 | 4.11 | 9.69 | 1.19 | 0.16 |
| Treatment delayed | Surgical and medical procedures | 10 | 9.60 | 2.64 | 34.87 | 9.59 | 17.76 | 1.58 | 0.47 |
| Pain in jaw | Musculoskeletal and connective tissue disorders | 9 | 4.32 | 1.54 | 12.13 | 4.32 | 9.17 | 1.22 | 0.14 |
| Pulmonary congestion | Respiratory, thoracic and mediastinal disorders | 9 | 25.91 | 3.28 | 204.50 | 25.89 | 21.54 | 1.80 | 0.60 |
| Procedural nausea | Injury, poisoning and procedural complications | 9 |  |  |  | 54.65 | 25.02 | 1.88 | 0.68 |
| Dyslalia | Nervous system disorders | 8 |  |  |  | 48.90 | 22.16 | 1.87 | 0.62 |
| Cardiac pacemaker insertion | Surgical and medical procedures | 7 | 6.72 | 1.74 | 25.97 | 6.71 | 10.21 | 1.44 | 0.19 |
| Oxygen therapy | Surgical and medical procedures | 7 | 10.07 | 2.09 | 48.49 | 10.07 | 12.71 | 1.59 | 0.31 |
| Stent placement | Surgical and medical procedures | 7 | 20.15 | 2.48 | 163.76 | 20.13 | 15.91 | 1.76 | 0.45 |
| Positive airway pressure therapy | Surgical and medical procedures | 6 | 8.63 | 1.74 | 42.78 | 8.63 | 10.12 | 1.54 | 0.20 |
| Pre-existing condition improved | General disorders and administration site conditions | 6 | 8.63 | 1.74 | 42.78 | 8.63 | 10.12 | 1.54 | 0.20 |
| Immobile | Social circumstances | 6 | 17.27 | 2.08 | 143.43 | 17.26 | 13.13 | 1.73 | 0.35 |
| Lumbar vertebral fracture | Injury, poisoning and procedural complications | 6 | 17.27 | 2.08 | 143.43 | 17.26 | 13.13 | 1.73 | 0.35 |
| Tooth extraction | Surgical and medical procedures | 6 | 17.27 | 2.08 | 143.43 | 17.26 | 13.13 | 1.73 | 0.35 |
| Dependence on respirator | Respiratory, thoracic and mediastinal disorders | 6 |  |  |  | 37.39 | 16.45 | 1.85 | 0.48 |
| Coma | Nervous system disorders | 5 | 7.19 | 1.40 | 37.08 | 7.19 | 7.62 | 1.47 | 0.05 |
| Hepatic failure | Hepatobiliary disorders | 5 | 7.19 | 1.40 | 37.08 | 7.19 | 7.62 | 1.47 | 0.05 |
| Metastases to liver | Neoplasms benign, malignant and unspecified | 5 | 7.19 | 1.40 | 37.08 | 7.19 | 7.62 | 1.47 | 0.05 |
| Loss of therapeutic response | General disorders and administration site conditions | 5 | 14.39 | 1.68 | 123.16 | 14.38 | 10.38 | 1.69 | 0.23 |
| Abscess limb | Infections and infestations | 4 | 11.51 | 1.29 | 102.98 | 11.51 | 7.68 | 1.63 | 0.07 |
| Angina pectoris | Cardiac disorders | 4 | 11.51 | 1.29 | 102.98 | 11.51 | 7.68 | 1.63 | 0.07 |
| Colostomy | Surgical and medical procedures | 4 | 11.51 | 1.29 | 102.98 | 11.51 | 7.68 | 1.63 | 0.07 |
| Endodontic procedure | Surgical and medical procedures | 4 | 11.51 | 1.29 | 102.98 | 11.51 | 7.68 | 1.63 | 0.07 |
| Face injury | Injury, poisoning and procedural complications | 4 | 11.51 | 1.29 | 102.98 | 11.51 | 7.68 | 1.63 | 0.07 |
| Tracheostomy | Surgical and medical procedures | 4 | 11.51 | 1.29 | 102.98 | 11.51 | 7.68 | 1.63 | 0.07 |
| Catheterisation cardiac | Investigations | 4 |  |  |  | 25.89 | 10.77 | 1.80 | 0.26 |
| Herpes zoster reactivation | Infections and infestations | 4 |  |  |  | 25.89 | 10.77 | 1.80 | 0.26 |
| Monoplegia | Nervous system disorders | 4 |  |  |  | 25.89 | 10.77 | 1.80 | 0.26 |
| Stress fracture | Injury, poisoning and procedural complications | 4 |  |  |  | 25.89 | 10.77 | 1.80 | 0.26 |
| Blood blister | Skin and subcutaneous tissue disorders | 3 |  |  |  | 20.13 | 7.96 | 1.76 | 0.11 |
| Blood glucose fluctuation | Investigations | 3 |  |  |  | 20.13 | 7.96 | 1.76 | 0.11 |
| Blood urea increased | Investigations | 3 |  |  |  | 20.13 | 7.96 | 1.76 | 0.11 |
| Cancer surgery | Surgical and medical procedures | 3 |  |  |  | 20.13 | 7.96 | 1.76 | 0.11 |
| Catarrh | Respiratory, thoracic and mediastinal disorders | 3 |  |  |  | 20.13 | 7.96 | 1.76 | 0.11 |
| Hallucination, auditory | Psychiatric disorders | 3 |  |  |  | 20.13 | 7.96 | 1.76 | 0.11 |
| Infected bite | Infections and infestations | 3 |  |  |  | 20.13 | 7.96 | 1.76 | 0.11 |
| Injection site haematoma | General disorders and administration site conditions | 3 |  |  |  | 20.13 | 7.96 | 1.76 | 0.11 |
| Intervertebral disc operation | Surgical and medical procedures | 3 |  |  |  | 20.13 | 7.96 | 1.76 | 0.11 |
| Post procedural haemorrhage | Injury, poisoning and procedural complications | 3 |  |  |  | 20.13 | 7.96 | 1.76 | 0.11 |
| Product dispensing issue | Injury, poisoning and procedural complications | 3 |  |  |  | 20.13 | 7.96 | 1.76 | 0.11 |
| Prostatitis | Reproductive system and breast disorders | 3 |  |  |  | 20.13 | 7.96 | 1.76 | 0.11 |
| Sinus operation | Surgical and medical procedures | 3 |  |  |  | 20.13 | 7.96 | 1.76 | 0.11 |

Abbreviation: PT: preferred terms; SOC: system organ categories; ROR: Reporting odds ratio; PRR: Proportional reporting ratio; IC: Information component; IC025: The lower limit of the 95%CI of IC.

Supplementary Table 7 PTs and corresponding SOCs with positive signals from all three methods for rozanolixizumab.

| **PT** | **SOC** | **N** | **ROR** | **95%CI lower** | **95%CI upper** | **PRR** | **Chi2** | **IC** | **IC025** |
| --- | --- | --- | --- | --- | --- | --- | --- | --- | --- |
| Myasthenia gravis | Nervous system disorders | 91 | 2.58 | 2.06 | 3.22 | 2.44 | 74.57 | 1.23 | 0.85 |
| Drug ineffective | General disorders and administration site conditions | 58 | 2.55 | 1.94 | 3.35 | 2.46 | 47.86 | 1.24 | 0.77 |
| Injection site pain | General disorders and administration site conditions | 36 | 30.27 | 19.21 | 47.69 | 29.24 | 517.86 | 3.99 | 2.86 |
| Product dose omission issue | Injury, poisoning and procedural complications | 24 | 4.65 | 3.02 | 7.15 | 4.56 | 58.82 | 2.04 | 1.20 |
| Weight increased | Investigations | 13 | 3.00 | 1.69 | 5.31 | 2.97 | 15.68 | 1.49 | 0.51 |
| Weight decreased | Investigations | 11 | 2.52 | 1.36 | 4.66 | 2.50 | 9.23 | 1.26 | 0.26 |
| Hospitalisation | Surgical and medical procedures | 11 | 2.81 | 1.51 | 5.22 | 2.79 | 11.70 | 1.41 | 0.37 |
| Wrong technique in product usage process | Injury, poisoning and procedural complications | 11 | 16.41 | 7.94 | 33.93 | 16.24 | 105.00 | 3.48 | 1.98 |
| Injection site pruritus | General disorders and administration site conditions | 10 | 32.80 | 13.62 | 78.97 | 32.49 | 152.67 | 4.07 | 2.41 |
| Syringe issue | Product issues | 8 | 65.47 | 19.68 | 217.79 | 64.97 | 168.02 | 4.48 | 2.68 |
| Device issue | Product issues | 7 | 14.30 | 5.87 | 34.84 | 14.21 | 59.85 | 3.35 | 1.69 |
| Injection site bruising | General disorders and administration site conditions | 7 | 22.89 | 8.70 | 60.26 | 22.74 | 85.62 | 3.79 | 2.05 |
| Incorrect dose administered | Injury, poisoning and procedural complications | 6 | 3.92 | 1.68 | 9.15 | 3.90 | 11.56 | 1.84 | 0.43 |
| Therapeutic response decreased | General disorders and administration site conditions | 6 | 4.66 | 1.98 | 10.99 | 4.64 | 15.02 | 2.07 | 0.61 |
| Intentional dose omission | Injury, poisoning and procedural complications | 6 | 8.52 | 3.46 | 20.97 | 8.47 | 31.40 | 2.79 | 1.18 |
| Needle issue | Product issues | 6 | 49.01 | 13.81 | 173.94 | 48.73 | 112.23 | 4.33 | 2.46 |
| Injection site swelling | General disorders and administration site conditions | 5 | 7.09 | 2.69 | 18.69 | 7.06 | 21.39 | 2.58 | 0.93 |
| Injection site rash | General disorders and administration site conditions | 5 | 10.88 | 3.95 | 29.98 | 10.83 | 33.47 | 3.07 | 1.32 |
| Injection site erythema | General disorders and administration site conditions | 4 | 3.62 | 1.29 | 10.19 | 3.61 | 6.80 | 1.74 | 0.16 |
| Respiratory disorder | Respiratory, thoracic and mediastinal disorders | 4 | 3.72 | 1.32 | 10.50 | 3.71 | 7.12 | 1.78 | 0.19 |
| Adverse drug reaction | General disorders and administration site conditions | 4 | 3.83 | 1.36 | 10.82 | 3.82 | 7.47 | 1.82 | 0.22 |
| Deep vein thrombosis | Vascular disorders | 4 | 7.67 | 2.58 | 22.83 | 7.64 | 18.71 | 2.67 | 0.90 |
| Injection site mass | General disorders and administration site conditions | 4 | 130.45 | 14.57 | 1168.18 | 129.94 | 102.37 | 4.74 | 2.72 |
| Viral infection | Infections and infestations | 3 | 3.76 | 1.14 | 12.43 | 3.75 | 5.43 | 1.79 | 0.06 |
| Back disorder | Musculoskeletal and connective tissue disorders | 3 | 5.43 | 1.60 | 18.45 | 5.41 | 9.26 | 2.26 | 0.44 |
| Bedridden | Social circumstances | 3 | 6.11 | 1.78 | 20.99 | 6.09 | 10.76 | 2.40 | 0.56 |
| Gastroenteritis viral | Infections and infestations | 3 | 6.11 | 1.78 | 20.99 | 6.09 | 10.76 | 2.40 | 0.56 |
| Plasmapheresis | Surgical and medical procedures | 3 | 8.14 | 2.29 | 28.90 | 8.12 | 14.99 | 2.74 | 0.84 |
| Product storage error | Injury, poisoning and procedural complications | 3 | 10.86 | 2.94 | 40.17 | 10.83 | 20.08 | 3.07 | 1.12 |
| Therapeutic response unexpected | General disorders and administration site conditions | 3 | 12.22 | 3.24 | 46.11 | 12.18 | 22.40 | 3.19 | 1.23 |
| Knee operation | Surgical and medical procedures | 3 | 16.29 | 4.07 | 65.22 | 16.24 | 28.62 | 3.48 | 1.48 |
| Injection site irritation | General disorders and administration site conditions | 3 | 48.87 | 8.16 | 292.79 | 48.73 | 56.11 | 4.33 | 2.25 |
| Injection site induration | General disorders and administration site conditions | 3 | 97.74 | 10.16 | 940.47 | 97.46 | 71.61 | 4.65 | 2.56 |

Abbreviation: PT: preferred terms; SOC: system organ categories; ROR: Reporting odds ratio; PRR: Proportional reporting ratio; IC: Information component; IC025: The lower limit of the 95%CI of IC.

Supplementary Table 8 The onset time of AEs with positive signals for five drugs.

| **Onset Time** | **Eculizumab** | **Ravulizumab** | **Zilucoplan** | **Efgartigimod** | **Rozanolixizumab** |
| --- | --- | --- | --- | --- | --- |
| **0-30 d** | Arthralgia | Back pain | Drug ineffective | Myasthenia gravis crisis | Headache |
|  | Escherichia urinary tract infection | Fatigue | Myasthenia gravis | Inappropriate schedule of product administration | Injection site erythema |
|  | Gastric cancer | Feeling abnormal | Respiratory disorder | Mechanical ventilation | Injection site swelling |
|  | Neck pain | Asthenia | Viral infection | Salivary hypersecretion | Myasthenia gravis |
|  | Pyelonephritis | General physical health deterioration | Injection site pain | Myasthenia gravis | Meningitis aseptic |
|  | SARS-CoV-2 test positive | Meningococcal infection | Syringe issue | Urinary tract infection | Diarrhoea |
|  | Suspected COVID-19 | Muscular weakness |  | Procedural headache | Vomiting |
|  |  | Thymoma malignant |  | Therapy cessation | Pyrexia |
|  |  | Arthritis |  | Dehydration | Nausea |
|  |  |  |  | Symptom recurrence | Herpes zoster |
|  |  |  |  | Treatment delayed | Rash |
|  |  |  |  | Metastases to liver | Intentional dose omission |
|  |  |  |  | Ill-defined disorder | Adverse drug reaction |
|  |  |  |  | Nephrolithiasis | Fluid retention |
|  |  |  |  | Therapy interrupted | Therapy interrupted |
|  |  |  |  | Blood urea increased |  |
|  |  |  |  | Injection site erythema |  |
|  |  |  |  | Herpes zoster reactivation |  |
|  |  |  |  | Cataract |  |
|  |  |  |  | Injection site reaction |  |
|  |  |  |  | Physical deconditioning |  |
|  |  |  |  | Lumbar vertebral fracture |  |
|  |  |  |  | Hospitalisation |  |
|  |  |  |  | Dysarthria |  |
|  |  |  |  | Musculoskeletal chest pain |  |
|  |  |  |  | Tooth extraction |  |
|  |  |  |  | Spinal operation |  |
|  |  |  |  | Loss of personal independence in daily activities |  |
|  |  |  |  | Immobile |  |
|  |  |  |  | Pre-existing condition improved |  |
|  |  |  |  | Therapeutic product effect decreased |  |
|  |  |  |  | Upper respiratory tract infection |  |
|  |  |  |  | Stent placement |  |
|  |  |  |  | Thymectomy |  |
|  |  |  |  | Blood blister |  |
|  |  |  |  | Therapy change |  |
|  |  |  |  | Dyslalia |  |
|  |  |  |  | Choking |  |
|  |  |  |  | Bulbar palsy |  |
|  |  |  |  | Coma |  |
|  |  |  |  | Infusion site extravasation |  |
|  |  |  |  | Respiratory syncytial virus infection |  |
|  |  |  |  | Therapeutic product ineffective |  |
| **31-60 d** | Arthralgia | Fatigue | Myasthenia gravis | Inappropriate schedule of product administration | Myasthenia gravis |
|  | Influenza like illness | Asthenia | Injection site pain | Dehydration | Headache |
|  |  | Feeling abnormal | Needle issue | Dyspnoea at rest | Injection site swelling |
|  |  |  | Drug ineffective | Myasthenia gravis | Injection site erythema |
|  |  |  | Product storage error | Bulbar palsy | Haemorrhage |
|  |  |  | Wrong technique in product usage process | Myasthenia gravis crisis | Localised infection |
|  |  |  | Weight decreased | Therapy cessation | Vomiting |
|  |  |  |  | Therapeutic product effect decreased | Nausea |
|  |  |  |  | Prostate cancer | Eating disorder |
|  |  |  |  |  | Joint swelling |
|  |  |  |  |  | Diarrhoea |
|  |  |  |  |  | Herpes zoster |
| **61-90 d** |  | Herpes simplex | Myasthenia gravis | Myasthenia gravis crisis |  |
|  |  | Meningococcal infection | Therapeutic response decreased | Urinary tract infection |  |
|  |  |  | Injection site pain | Hallucination, auditory |  |
|  |  |  | Drug ineffective | Choking |  |
|  |  |  |  | Hospitalisation |  |
| **≥ 90 d** | Suspected COVID-19 | Visual impairment | Drug ineffective | Myasthenia gravis crisis | Myasthenia gravis |
|  | Mastication disorder | Therapeutic response shortened | Injection site erythema | Hospitalisation | Therapy interrupted |
|  | Neuromyelitis optica spectrum disorder | Photophobia | Injection site pain | Ill-defined disorder | Insurance issue |
|  | Memory impairment | Asthenia | Injection site induration | Myasthenia gravis | Nausea |
|  | Escherichia urinary tract infection | Dropped head syndrome | Incorrect dose administered | Urinary tract infection | Vomiting |
|  |  | Speech disorder | Myasthenia gravis | Dehydration | Plasmapheresis |
|  |  | Neck pain | Injection site pruritus | Cardiac failure congestive | Pyrexia |
|  |  | Nocturnal dyspnoea | Bedridden | Respiratory syncytial virus infection | Headache |
|  |  | Muscular weakness | Respiratory disorder | Choking | Syncope |
|  |  | Gait disturbance |  | Catarrh | Influenza like illness |
|  |  | Stress |  | Pulmonary congestion |  |
|  |  | Fatigue |  | Lacrimation increased |  |
|  |  | Eyelid ptosis |  | Therapy change |  |
|  |  | Meningococcal infection |  | Therapy cessation |  |
|  |  | Somnolence |  | Dysarthria |  |
|  |  | General physical health deterioration |  | Inappropriate schedule of product administration |  |
|  |  | Symptom recurrence |  | Surgery |  |
|  |  | Eye disorder |  | Upper respiratory tract infection |  |
|  |  |  |  | Respiratory tract infection |  |
|  |  |  |  | Symptom recurrence |  |
|  |  |  |  | Therapeutic product effect decreased |  |
|  |  |  |  | Intervertebral disc operation |  |
|  |  |  |  | Cancer surgery |  |
|  |  |  |  | Catheterisation cardiac |  |
|  |  |  |  | Therapy interrupted |  |
|  |  |  |  | Blepharospasm |  |
|  |  |  |  | Cataract |  |

Supplementary Table 9 Statistics of PTs for the death reports of five drugs.

| **Eculizumab** | | **Ravulizumab** | | **Zilucoplan** | | **Efgartigimod** | | **Rozanolixizumab** | |
| --- | --- | --- | --- | --- | --- | --- | --- | --- | --- |
| **PTs** | **N** | **PTs** | **N** | **PTs** | **N** | **PTs** | **N** | **PTs** | **N** |
| Death | 102 | Death | 54 | Death | 3 | Death | 130 | Death | 2 |
| Myasthenia gravis | 14 | Cardiac arrest | 3 | Drug ineffective | 3 | Myasthenia gravis crisis | 26 | Acute kidney injury | 2 |
| Pneumonia | 9 | Myasthenia gravis | 3 | Decreased appetite | 2 | Myasthenia gravis | 19 | Acute respiratory distress syndrome | 1 |
| Suspected COVID-19 | 8 | Myocardial infarction | 3 | Hospitalisation | 2 | Pneumonia | 17 | Acute respiratory failure | 1 |
| Myasthenia gravis crisis | 7 | Neoplasm malignant | 3 | Myasthenia gravis | 2 | Urinary tract infection | 16 | Cardiac failure | 1 |
| Dyspnoea | 6 | Respiratory failure | 3 | Pneumonia | 2 | Dyspnoea | 15 | Clostridium difficile infection | 1 |
| Sepsis | 6 | Sepsis | 3 | Acute kidney injury | 1 | COVID-19 | 14 | Colonic abscess | 1 |
| Drug ineffective | 5 | Lung neoplasm malignant | 2 | Acute pulmonary oedema | 1 | Fall | 13 | Diverticulitis | 1 |
| Fatigue | 5 | Off label use | 2 | Alanine aminotransferase increased | 1 | Sepsis | 12 | Dyspnoea | 1 |
| Myocardial infarction | 5 | Thymoma malignant | 2 | Anaemia macrocytic | 1 | Symptom recurrence | 12 | Localised infection | 1 |

Abbreviation: PT: preferred terms.
